# An expanded mammal mitogenome dataset from Southeast Asia

**DOI:** 10.1093/gigascience/gix053

**Published:** 2017-07-13

**Authors:** Faezah Mohd Salleh, Jazmín Ramos-Madrigal, Fernando Peñaloza, Shanlin Liu, S. Sinding Mikkel-Holger, P. Patel Riddhi, Renata Martins, Dorina Lenz, Jörns Fickel, Christian Roos, Mohd Shahir Shamsir, Mohammad Shahfiz Azman, K. Lim Burton, J. Rossiter Stephen, Andreas Wilting, M. Thomas P. Gilbert

**Affiliations:** 1 Centre for GeoGenetics, Natural History Museum of Denmark, University of Copenhagen, Øster Voldgade 5-7, 1350, Copenhagen, Denmark; 2 Faculty of Biosciences and Medical Engineering, Universiti Teknologi Malaysia, 81310 Johor Bahru, Johor, Malaysia; 3 Leibniz Institute for Zoo and Wildlife Research, Alfred-Kowalke Strasse 17, 10315 Berlin, Germany; 4 Undergraduate Program on Genomic Sciences, Universidad Nacional Autonoma de Mexico, 62210 Cuernavaca, Mexico; 5 BGI-Shenzhen, Shenzhen, GuangDong, China; 6 Natural History Museum, University of Oslo, PO Box 1172 Blindern, NO-0318, Oslo, Norway; 7 Freie Universität Berlin, Kaiserswerther Str. 16-18, 14195 Berlin, Germany; 8 University of Potsdam, Institute for Biochemistry and Biology, Karl-Liebknecht-Str 24-25, 14476 Potsdam, Germany; 9 Gene Bank of Primates and Primate Genetics Laboratory, German Primate Center, Leibniz Institute for Primate Research, Kellnerweg 4, 37077, Göttingen, Germany; 10 Forest Biodiversity Division, Forest Research Institute Malaysia, 52109 Kepong, Selangor, Malaysia; 11 Department of Natural History, Royal Ontario Museum, Toronto, Canada; 12 School of Biological and Chemical Sciences, Queen Mary University of London, Mile End Road, London E1 4NS, United Kingdom; 13 NTNU University Museum, Norwegian University of Science and Technology, Trondheim, Norway

**Keywords:** invertebrate-derived (iDNA), metabarcoding, GenBank, taxonomic assignment

## Abstract

Southeast (SE) Asia is 1 of the most biodiverse regions in the world, and it holds approximately 20% of all mammal species. Despite this, the majority of SE Asia's genetic diversity is still poorly characterized. The growing interest in using environmental DNA to assess and monitor SE Asian species, in particular threatened mammals—has created the urgent need to expand the available reference database of mitochondrial barcode and complete mitogenome sequences. We have partially addressed this need by generating 72 new mitogenome sequences reconstructed from DNA isolated from a range of historical and modern tissue samples. Approximately 55 gigabases of raw sequence were generated. From this data, we assembled 72 complete mitogenome sequences, with an average depth of coverage of ×102.9 and ×55.2 for modern samples and historical samples, respectively. This dataset represents 52 species, of which 30 species had no previous mitogenome data available. The mitogenomes were geotagged to their sampling location, where known, to display a detailed geographical distribution of the species. Our new database of 52 taxa will strongly enhance the utility of environmental DNA approaches for monitoring mammals in SE Asia as it greatly increases the likelihoods that identification of metabarcoding sequencing reads can be assigned to reference sequences. This magnifies the confidence in species detections and thus allows more robust surveys and monitoring programmes of SE Asia's threatened mammal biodiversity. The extensive collections of historical samples from SE Asia in western and SE Asian museums should serve as additional valuable material to further enrich this reference database.

## Data Description

### Context

Southeast (SE) Asia is 1 of the most biodiverse regions in the world, hosting ∼20% of mammal species, but it is experiencing rapid deforestation for agriculture and development. To assess the ecological consequences of land use change, there is growing interest in using environmental DNA to monitor mammal populations, particularly threatened taxa that often underpin conservation policies [1–4]. Yet current efforts are hampered by the lack of a reference database of mitochondrial barcodes and complete mitogenome sequences. Currently there are 922 mammalian mitogenomes available in Genbank. Unfortunately, most are not tagged by location/origin. Data mining through manual screening of each mitogenomes resulted in 174 terrestrial mammal species that are typical to SE Asia. In this work, 30 novel species are added, contributing to ∼17% expansion of the current SE Asia mammal mitogenome database.

### DNA extraction

Genomic DNA was extracted from different sample types of 72 small mammals, comprising 52 species, listed in Table 1 and Table 2. DNA from modern tissue and blood samples was isolated using the Qiagen DNeasy extraction kit (Qiagen, Hilden, Germany, [QIAGEN, RRID:SCR_008539]) or Invitek DNA extraction kit (Invitek GmbH, Berlin, Germany), as per standard protocols following the manufacturer's guidelines. Historical samples obtained from the Zoological Museum, Natural History Museum of Denmark, and University of Copenhagen (ZM, KU) were treated differently according to type of tissue (Additional file 1a), while at the German Primate Center, DNA extraction from museum specimens followed Liedigk et al. (2015) [5] using the Gen-IAL First All Tissue Kit (Gen-IAL, Troisdorf, Germany). Complete details of sample information are provided in Additional file 2.

**Table 1:** List of mitogenomes assembled in this work that supplement preexisting mitogenome references already available in GenBank

No.	GenBank ID	Common name	Genus	Species	Assembly size	Locality	Source	Sample date of collection	Data by
1	KY117537	Hog deer	*Axis*	*porcinus*	16 402	CPH Zoo	ZM, KU	21/8/1912	F.M.S./F.P.
2	KY117538	Pallas's squirrel	*Callosciurus*	*erythraeus*	16 656	Bangkok, Thailand	ZM, KU	25/5/1969	F.M.S./F.P.
3	KX265095	Bay cat	*Catopuma*	*badia*	16 960	Sabah, Malaysia	National Museum Scotland	20/04/2000	P.R.P.
4	KX224524	Asiatic golden cat	*Catopuma*	*temminckii*	16 960	Thailand	American Museum of National History, New York.	10/10/1927	P.R.P.
5	KY117545	Sumatran rhino	*Dicerorhinus*	*sumatrensis*	16 466	Sumatra, Indonesia	Naturalis, Leiden, The Netherlands	1880	R.M.
6	KY117546	Least pygmy squirrel	*Exilisciurus*	*exilis*	16 637	Indonesia	ROM	16/06/1993	F.M.S./F.P.
7	KY117548	Hose's mongoose	*Herpestes*	*javanicus*	16 340	Java, Indonesia	ZM, KU	12/3/1947	F.M.S./F.P.
8	KY117550	Three-striped ground Squirrel	*Lariscus*	*indsignis*	16 399	Maybe Malaysia	ZM, KU	Unknown	F.M.S./F.P.
9	KY117592	Black crested macaque	*Macaca*	*nigra*	16 558	Captive	Gettorf Zoo, Germany	18/07/2000	C.R.
10	KY117593	Northern pig-tailed macaque	*Macaca*	*leonina*	16 554	Captive	Ludwig-Maximilans-University Munich, Germany	6/3/1995	C.R.
11	KY117594	Southern pig-tailed macaque	*Macaca*	*nemestrina*	16 531	Peninsular Malaysia	National Museum Scotland, Edinburgh, UK	Unknown	C.R.
12	KT288227	Marbled cat	*Pardofelis*	*marmorata*	17 218	Sumatra, Indonesia	National Archaeological Museum of the Netherlands, Leiden	30/08/1930	P.R.P.
13	KY117602	Sumatra surili	*Presbytis*	*melalophos*	16 558	Captive	Howletts Wild Animal Park, UK	23/7/1999	C.R.
14	KR135743	Flat-headed cat	*Prionailurus*	*planiceps*	17 704	Sabah, Malaysia	Sabah Wildlife Department	25/04/2000	P.R.P.
15	KY117580	Malayan field rat	*Rattus*	*tiomanicus*	16 415	SPF Bidor, Perak, Malaysia	FRIM	12/2/2011	F.M.S./F.P.
16	KY117579	Malayan field rat	*Rattus*	*tiomanicus*	16 312	Indonesia	ROM	01/06/1993	F.M.S./F.P.
17	KY117581	Malayan field rat	*Rattus*	*tiomanicus*	16 305	Hutan Simpan Chikus, Tapah Perak, Malaysia	FRIM	13/1/2011	F.M.S./F.P.
19	KY117582	Black giant squirrel	*Ratufa*	*bicolor peninsulae*	16 600	Bang Nara, Malakka, Thailand	ZM, KU	3/12/1932	F.M.S./F.P.
18	KY117574	Javan rhino	*Rhinoceros*	*sondaicus*	16 417	Java, Indonesia	Copenhagen Natural History Museum	Unknown	R.M.
20	KY117575	Javan rusa	*Rusa*	*timorensis*	16 437	Toeloeng Agoeng, West Java, Indonesia	Naturalis, Leiden, The Netherlands	Unknown	R.M.
21	KY117576	Indian sambar deer	*Rusa*	*unicolor dejeani*	16 437	Mentawai, Indonesia	Naturalis, Leiden, The Netherlands	Unknown	R.M.
22	KY117599	Western purple-faced langur	*Semnopithecus*	*vetulus*	16 545	Captive	Belfast Zoo, UK	9/11/1998	C.R.
23	KY117589	Malayan tapir	*Tapirus*	*indicus*	16 794	Captive	Copenhagen Zoo	11/1/2015	F.M.S./F.P.
24	KY117598	Silvered langur	*Trachypithecus*	*cristatus*	16 551	North Sumatra, Indonesia	Bavarian State Collection Munich, Germany	1911	C.R.

FRIM: Forest Research Institute, Malaysia; ROM: Royal Ontario Museum; ZM, KU: Zoological Museum, University of Copenhagen.

**Table 2:** List of mitogenomes assembled in this work that have no previous complete mitogenome reference available in GenBank

No.	GenBank ID	Common name	Genus	Species	Assembly size	Locality	Source	Sample date of collection	Data by
1	KY117536	Asian small-clawed otter	*Aonyx*	*cinereus*	16 153	Captive	Copenhagen Zoo	08/08/11	F.M.S./F.P.
2	KY117535	Asian small-clawed otter	*Aonyx*	*cinereus*	16 153	Sarawak, Malaysia	British Museum of Natural History, London	25/8/2010	F.M.S./F.P.
3	KY117560	Binturong	*Arctictis*	*binturong*	17 067	Unknown	Tierpark, Berlin	29/11/2010	P.R.P.
4	KY117541	Plantain squirrel	*Callosciurus*	*notatus*	16 582	Hutan Bidor, Perak, Malaysia	FRIM	11/2/2011	F.M.S./F.P.
5	KY117542	Plantain squirrel	*Callosciurus*	*notatus*	16 599	East Kalimantan, Indonesia	ROM	03/06/1993	F.M.S./F.P.
6	KY117543	Prevost's squirrel	*Callosciurus*	*prevostii*	16 674	East Kalimantan, Indonesia	ROM	15/06/1993	F.M.S./F.P.
7	KY117540	Variable squirrel	*Callosciurus*	*finlaysonii frandseni*	15 747	Koh Chang, Thailand	ZM, KU	14/1/1900	F.M.S./F.P.
8	KY117539	Variable squirrel	*Callosciurus*	*finlaysonii*	16 489	Central Thailand	ZM, KU	2/2/1928	F.M.S./F.P.
9	KY117544	Sunda otter civet	*Cynogale*	*bennetti*	15 784	Borneo	British Museum of Natural History, London	25/8/2010	F.M.S./F.P.
10	KY117549	Greater mouse deer	*Tragulus*	*napu*	15 778	Bang Nara, Thailand	ZM, KU	11/10/1931	F.M.S./F.P.
11	KY117552	Long-tailed giant rat	*Leopaldamys*	*sabanus*	15 973	G. Telapak Buruk, Negeri Sembilan, Malaysia	FRIM	24/2/2010	F.M.S./F.P.
12	KY117553	Long-tailed giant rat	*Leopaldamys*	*sabanus*	15 972	Teluk Segadas, P. Pangkor, Perak, Malaysia	FRIM	19/3/2010	F.M.S./F.P.
13	KY117554	Long-tailed giant rat	*Leopaldamys*	*sabanus*	15 974	Hutan Simpan Temengor, Gerik Perak, Malaysia	FRIM	23/1/2014	F.M.S./F.P.
14	KY117555	Long-tailed giant rat	*Leopaldamys*	*sabanus*	15 972	Hutan Simpan Lenggor, Kluang, Johor, Malaysia	FRIM	19/2/2014	F.M.S./F.P.
15	KY117551	Long-tailed giant rat	*Leopaldamys*	*sabanus*	15 974	Malaysia	ROM	28/05/1993	F.M.S./F.P.
16	KY117556	Hairy-nosed otter	*Lutra*	*sumatrana*	16 580	Bang Nara, Thailand	ZM, KU	1/4/1939	F.M.S./F.P.
17	KY117557	Smooth-coated otter	*Lutrogale*	*perspicillata*	16 042	Melaka, Malaysia	British Museum of Natural History, London	25/8/2010	F.M.S./F.P.
18	KY117558	Smooth-coated otter	*Lutrogale*	*perspicillata*	16 041	Bang Nara, Thailand	ZM, KU	24/1/1933	F.M.S./F.P.
19	KY117591	Moor macaque	*Macaca*	*maura*	16 563	Captive	Hannover Zoo, Germany	20/8/1998	C.R.
20	KY117564	Rajah/brown spiny rat	*Maxomys*	*rajah*	16 200	Indonesia	ROM	06/06/1993	F.M.S./F.P.
21	KY117562	Rajah/brown spiny rat	*Maxomys*	*rajah*	16 296	Teluk Segadas, P. Pangkor, Perak, Malaysia	FRIM	19/3/2010	F.M.S./F.P.
22	KY117563	Rajah/brown spiny rat	*Maxomys*	*rajah*	16 296	Pasir Bogak, P.Pangkor, Perak, Malaysia	FRIM	18/3/2010	F.M.S./F.P.
23	KY117567	Red spiny rat	*Maxomys*	*surifer*	16 286	50 ha, Pasoh, Negeri Sembilan, Malaysia	FRIM	12/6/2008	F.M.S./F.P.
24	KY117566	Red spiny rat	*Maxomys*	*surifer*	16 290	Indonesia	ROM	21/05/1993	F.M.S./F.P.
25	KY117565	Red spiny rat	*Maxomys*	*surifer*	16 286	Malaysia	ROM	17/05/2013	F.M.S./F.P.
26	KY117570	Whitehead's spiny rat	*Maxomys*	*whiteheadi*	16 316	Hutan Simpan Bikam, Perak, Malaysia	FRIM	12/2/2011	F.M.S./F.P.
27	KY117571	Whitehead's spiny rat	*Maxomys*	*whiteheadi*	16 316	Keruing Trail, FRIM, Kepong, Selangor, Malaysia	FRIM	13/3/2013	F.M.S./F.P.
28	KY117568	Whitehead's spiny rat	*Maxomys*	*whiteheadi*	16 287	Hutan Simpan Bikam, Perak, Malaysia	FRIM	12/2/2011	F.M.S./F.P.
29	KY117569	Whitehead's spiny rat	*Maxomys*	*whiteheadi*	16 429	Bukit Tapah, Perak, Malaysia	FRIM	23/3/2011	F.M.S./F.P.
30	KY052142	Indian muntjac	*Muntiacus*	*muntjak*	16 354	West Java, Indonesia	Vienna NHM	1858	R.M.
31	KY117559	Bornean yellow muntjac	*Muntiacus*	*atherodes*	16 354	Koemai, West Borneo	Bonn NHM	1938	R.M.
32	KY117573	Dark-tailed tree rat	*Niviventer*	*cremoriventer*	16 322	Track 5 (G.Inas), Kedah, Malaysia	FRIM	5/11/2009	F.M.S./F.P.
33	KY117572	Dark-tailed tree rat	*Niviventer*	*cremoriventer*	16 234	Malaysia	ROM	17/05/2013	F.M.S./F.P.
34	KY117600	Grizzled leaf monkey	*Presbytis*	*comata comata*	16 551	Captive	Howletts Wild Animal Park, UK	23/12/1999	C.R.
35	KY117601	Mitred leaf monkey	*Presbytis*	*mitrata*	16 555	Captive	Howletts Wild Animal Park, UK	12/11/1998	C.R.
36	KX857784	Leopard cat	*Prionailurus*	*bengalensis*	16 989	Thailand	American Museum of National History, New York.	25/02/1924	P.R.P.
37	KY117578	Annandale's sundaic rat	*Rattus*	*annandalei*	16 297	Hutan Simpan Bikam, Perak, Malaysia	FRIM	12/2/2011	F.M.S./F.P.
38	KY117577	Annandale's sundaic rat	*Rattus*	*annandalei*	16 301	Hutan Simpan Bikam, Perak, Malaysia	FRIM	11/2/2011	F.M.S./F.P.
39	KY117583	Mountain giant sunda rat	*Sundamys*	*infraluteus*	16 297	Malaysia	ROM	18/05/2013	F.M.S./F.P.
40	KY117585	Müller's giant sunda rat	*Sundamys*	*meulleri*	16 326	Track 1 (G.Inas), Kedah, Malaysia	FRIM	5/11/2009	F.M.S./F.P.
41	KY117584	Müller's giant sunda rat	*Sundamys*	*meulleri*	16 304	Malaysia	ROM	01/06/2013	F.M.S./F.P.
42	KY117586	Brooke's squirrel	*Sundasciurus*	*brookei*	16 417	East Kalimantan, Indonesia	ROM	13/06/1993	F.M.S./F.P.
43	KY117587	Low's squirrel	*Sundasciurus*	*lowii*	16 307	East Kalimantan, Indonesia	ROM	06/06/1993	F.M.S./F.P.
44	KY117588	Low's squirrel	*Sundasciurus*	*sp*	16 458	East Kalimantan, Indonesia	ROM	21/06/1993	F.M.S./F.P.
45	KY117595	Phayre's langur	*Trachypithecus*	*phayrei phayrei*	16 548	South West Myanmar	Natural History Museum Berlin, Germany	Unknown	C.R.
46	KY117596	East Javan ebony langur	*Trachypithecus*	*auratus*	16 552	Captive	Bristol Zoo, UK	26/10/2010	C.R.
47	KY117597	West Javan ebony langur	*Trachypithecus*	*mauritius*	16 554	West Java, Indonesia	Naturalis Leiden; Netherlands	Unknown	C.R.
48	KY117590	Long-tailed porcupine	*Trichys*	*fasciculata*	16 328	Borneo	ZM, KU	5/10/1894	F.M.S./F.P.

FRIM: Forest Research Institute, Malaysia; NHM: Natural History Museum; ROM: Royal Ontario Museum; ZM, KU: Zoological Museum, University of Copenhagen.

## Data Validation and Quality Control

### Mitogenome sequencing, assembly, and annotation

Mitogenomes were generated using several approaches. In Copenhagen, author F.M.S. constructed Illumina shotgun libraries with insert sizes ranging between 50 and 400 bp. To construct libraries, DNA was sheared to the target size range using Bioruptor® XL (Diagenode, USA [Diagenode, RRID:SCR_014807]) and converted into an Illumina-compatible sequencing library using the NEBNext E6070 Kit (New England Biolabs, UK). The libraries were polymerase chain reaction (PCR) amplified with index primers and purified using Qiaquick columns (Qiagen, Hilden, Germany) according to the manufacturer's instruction (Additional file 1b). Multiple libraries were combined together into 3 pools, normalized to 10 nM, and sequenced across 3 lanes of Illumina HiSeq 2500 using SR100 bp chemistry. In Berlin and Goettingen, mitogenomes were generated by authors P.R.P. and C.R. using overlapping PCR products using long-range PCR (Additional file 1c) followed by library construction and MiSeq sequencing, or Sanger sequencing as described in Patel, Förster, and Kitchener (2016) [6] and Liedigk et al. (2015), Roos et al. (2011), and Liedigk et al. (2014) [5, 7, 8], respectively. Author R.M.’s mitogenomes were done using methods outlined in Fortes and Paijmans (2015) [9]. Further details about laboratory methods are described in Additional file 1.

Raw reads for F.M.S. samples were assembled independently by authors F.M.S. and F.P. using 2 different approaches, then compared for consistency. Author F.M.S. trimmed the reads for sequencing adapters, low-quality stretches, and leading/tailing Ns using AdapterRemoval 1.2 (AdapterRemoval, RRID:SCR_011834) [10]. The mitochondrial genome was reconstructed with MITObim v. 1.8 [11] using the reference mitogenome of the closest species available in GenBank as the seed reference (Additional file 2). In order to obtain the mapping statistics of the samples, we ran PALEOMIX v. 1.2.6 [12] with default parameters where reads shorter than 25 bp after trimming were discarded. The trimmed reads were aligned against the newly assembled mitogenome generated by MITObim using Burrows–Wheeler Aligner [13]. Alignments showing low-quality scores and PCR duplicates were further removed using the MarkDuplicates program from Picard tools, and reads were locally realigned around small insertions and deletions (indels) to improve overall genome quality using the IndelRealigner tool from the Genome Analysis Toolkit (GATK, RRID:SCR_001876) [14]. In contrast, author F.P. inputted the trimmed reads into mitoMaker [15], which performs a *de novo* and reference-based assembly using SOAPdenovoTrans v. 1.03 (SOAPdenovo-Trans, RRID:SCR_013268) [16] and MITObim v. 1.7 [11]. Post-assembly, the F.M.S. and F.P. mitogenomes were manually compared for consistency by F.M.S. to generate the final consensus sequences. These assemblies were automatically annotated using tRNAscan-SE v. 1.4 (tRNAscan-SE, RRID:SCR_010835) [17] and Basic Local Alignment Search Tool v. 2.2.29 (NCBI BLAST, RRID:SCR_004870) [18] using the mitochondrial genomes found in the National Center for Biotechnology Information Reference Sequence Database (RefSeq, RRID:SCR_003496) [19] as references.

For the mitogenome constructed by author R.M., Illumina sequence reads were de-multiplexed according to the respective indexes with the Illumina software bcl2fastq v. 2.17 (Illumina, San Diego, CA, USA), and adapters were clipped from the sequence reads with the software cutadapt v. 1.3 [20]. Quality trimming was done through a sliding window approach (10 bp; Q20), and all reads shorter than 20 bp were removed from the analyses. Mitogenome references from target or closely related species were used for mapping of the sequencing reads. Aligned reads were de-duplicated using MarkDuplicates from Picard-tools v. 1.106 (Picard, RRID:SCR_006525) [21]. VariantCalling was carried out using Samtools v. 1.1 (SAMTOOLS, RRID:SCR_002105) [13] and Bcftools v. 1.2 (SAMtools/BCFtools, RRID:SCR_005227) [22]. For each sample, GATK [14] variant calling output files were further filtered to have a minimum read coverage ≥ ×3, and variants were only called when the corresponding base was represented by ≥50%; otherwise this position was “N”-masked.

**Figure 1:**
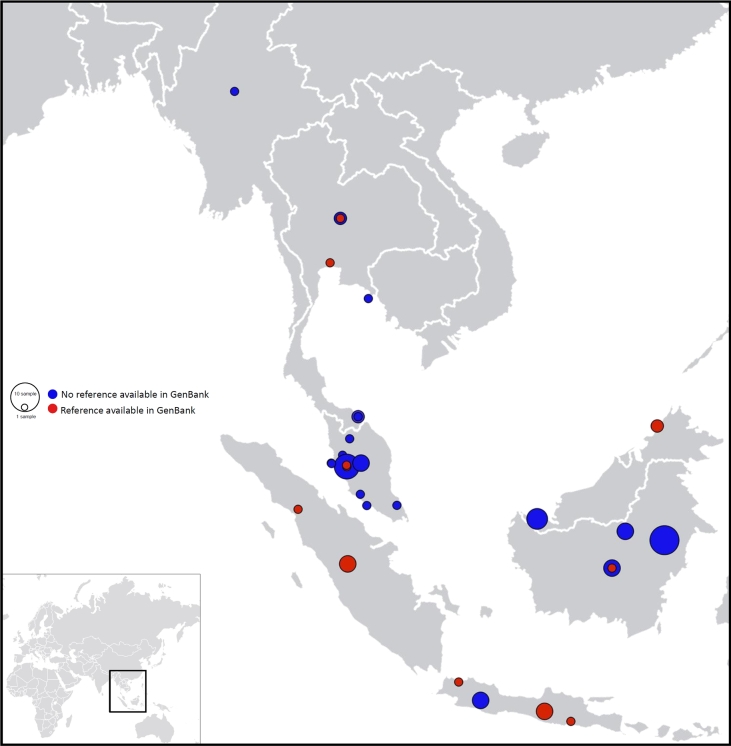
Geographical distribution of mitogenomes assembled in this work (60 mitogenomes with known locality).

Numbers of raw reads generated for each sample and mapping statistics for all 72 mitogenome assemblies are shown in Additional file 2. Sanger sequenced mitogenomes were checked with 4Peaks 1.8 (4Peaks, RRID:SCR_000015) [23], assembled with SeaView 4.5.4 [24], and annotated with DOGMA [25]. All mitogenomes were checked manually by eye to identify possible errors caused by insertion and deletions in Tablet [26]. The final mitochondrial genomes have been uploaded to GenBank (accession numbers are provided in Tables 1 and 2). The details of all new mitogenomes assembled in this work are given in Tables 1 and 2. Mitogenomes (60 samples) with known localities were geotagged and mapped to display their geographical distribution (Fig. 1).

### Phylogenetic analysis

**Figure 2:**
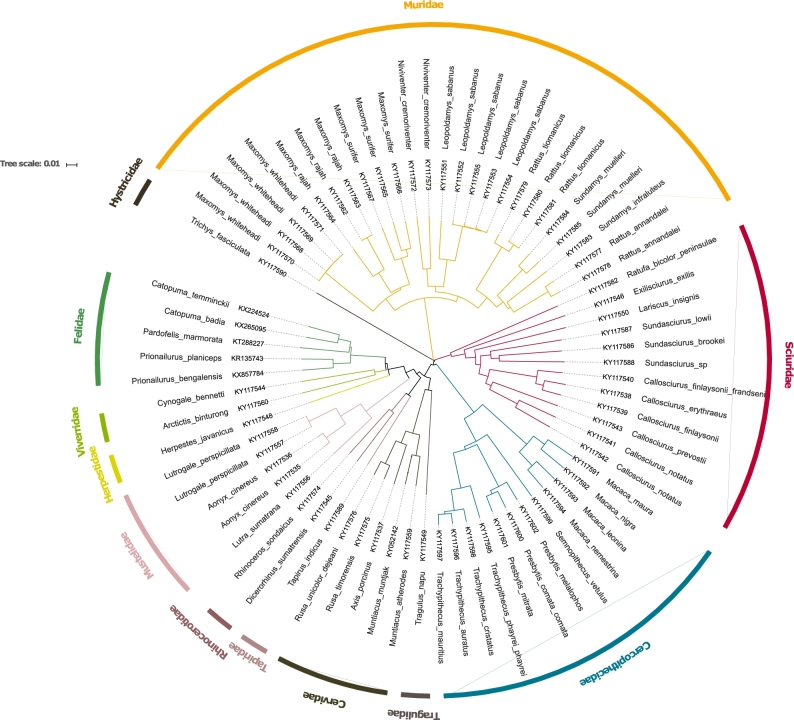
Phylogenetic tree of mitogenomes assembled in this work.

All the sequenced mitogenomes were aligned using MAFFT v. 7.158b (MAFFT, RRID:SCR_011811) [27] using the E-INS-i option (Additional file 3). Randomized Axelerated Maximum Likelihood (RAxML) v. 8.0.26 (RAxML, RRID:SCR_006086) [28] was used to perform the phylogenetic analysis with a GTR+GAMMA model of nucleotide substitution. To obtain node support, we used 100 bootstrap pseudo-replicates (Fig. 2). The newick file is provided as Additional file 4.

## Re-use Potential

We anticipate that the now-expanded mitogenome reference dataset for SE Asian mammals will provide benefits for a number of research areas. First, it should enhance the power of environmental DNA and other metabarcoding/barcoding approaches that relate to the identification of SE Asian mammals by conferring the ability to identify more species to the species level. This in turn has practical applications for those monitoring SE Asia's threatened mammal biodiversity, combatting trade in mammal species and so on. Second, the data will also have relevance to phylogenetic and population studies based on mtDNA data, which will be of use as we investigate the evolutionary history of this biodiversity hotspot.

## Availability of supporting data

Raw shotgun data are deposited in the SRA under bioproject number PRJNA361218 and are available in the *GigaScience* repository, GigaDB [29]. Details of the method to support this work can be found in protocols.io [30].

## Additional files

1. Additional file 1: DNA extraction of historical samples, library construction, and primer information

2. Additional file 2: Sample information sheet of mitogenomes assembled in this work

3. Additional file 3: Alignment of mitogenomes assembled in this work

4. Additional file 4: Newick file for phylogenetic tree

## Abbreviations

BLAST: Basic Local Alignment Search Tool; bp: base pair; GATK: Genome Analysis Toolkit; MAFFT: Multiple Alignment using Fast Fourier Transform; NCBI RefSeq: National Center for Biotechnology Information Reference Sequence Database; PCR: polymerase chain reaction; RAxML: Randomized Axelerated Maximum Likelihood; SE: southeast.

## Competing interests

The authors declare that they have no competing interests.

## Funding

This project was funded by the Malaysian Government (F.M.S.), Lundbeck Foundation grant R52–5062 (M.T.P.G.), Leibniz-Association grant SAW-2013-IZW-2 (J.F.), the German Federal Ministry of Education and Research grant BMBF FKZ: 01LN1301A (A.W.), and the German Primate Center (C.R.).

## Author contributions

F.M.S., A.W., J.F., and M.T.P.G. conceived the project. F.M.S., M.H.S.S., M.S.S., M.S.A., R.M., P.R.P., C.R., B.K.L., and S.J.R. collected the samples and extracted the genomic DNA. F.M.S., R.M., P.R.P., and C.R. constructed the libraries and did sequencing. F.M.S., J.R.M., F.P., S.L., P.R.P., R.M., D.L., and C.R. assembled the mitogenomes and performed mitogenome analysis. F.M.S., S.L., P.R.P., and M.T.P.G. wrote the article. All authors discussed the project and data. All authors read and approved the final manuscript.

## Supplementary Material

GIGA-D-17-00015_Original-Submission.pdf

GIGA-D-17-00015_Revision-1.pdf

GIGA-D-17-00015_Revision-2.pdf

Response-to-Reviewer-Comments_Original-Submission.pdf

Response-to-Reviewer-Comments_Revision-1.pdf

Reviewer-1-Report-(Original-Submission).pdf

Reviewer-1-Report-(Revision-1).pdf

Reviewer-2-Report-(Original-Submission).pdf

Additional-files
