# Supplementary material for: An expanded mammal mitogenome dataset from Southeast Asia
Source: Gigascience. 2017 Jul 13;6(8):1–8. doi: 10.1093/gigascience/gix053 (PMC5737531; doi:10.1093/gigascience/gix053)
Supplement: GIGA-D-17-00015_Original-Submission.pdf [file gix053_giga-d-17-00015_original-submission.pdf]

Manuscript
Click here to download Manuscript 1ManuscriptmaintextSallehetal_updated 140217.docx

Title: An expanded mammal mitogenome dataset from Southeast Asia

Faezah Mohd Salleh[1,2*], Jazmín Ramos-Madrigal[1], Fernando Peñaloza[3,4], Shanlin Liu[1,5], Mikkel-Holger S. Sinding[1,6], Riddhi P. Patel[3,7], Renata Martins[3], Dorina Lenz[3], Christian Roos[8], Mohd Shahir Shamsir[2], Mohammad Shahfiz Azman[9], Burton K. Lim[10], Stephen J. Rossiter[11], Andreas Wilting[3], M. Thomas P. Gilbert[1,12*]

*Correspondence: faezah@fbb.utm.my and tgilbert@snm.ku.dk

[1]Centre for GeoGenetics, Natural History Museum of Denmark, University of Copenhagen, Øster Voldgade 5-7, 1350, Copenhagen, Denmark

[2]Faculty of Biosciences and Medical Engineering, Universiti Teknologi Malaysia, 81310 Johor Bahru, Johor, Malaysia

[3]Leibniz Institute for Zoo and Wildlife Research, Alfred-Kowalke Strasse 17, 10315 Berlin, Germany.

[4]Undergraduate Program on Genomic Sciences, Universidad Nacional Autonoma de Mexico, 62210 Cuernavaca, Mexico

[5]BGI-Shenzhen, Shenzhen, GuangDong, China

[6]Natural History Museum, University of Oslo, P.O. Box 1172 Blindern, NO-0318, Oslo, Norway

[7]Freie Universität Berlin, Kaiserswerther Str. 16-18, 14195 Berlin, Germany

[8]Gene Bank of Primates and Primate Genetics Laboratory, German Primate Center, Leibniz Institute for Primate Research, Kellnerweg 4, 37077, Göttingen, Germany

[9]Forest Biodiversity Division, Forest Research Institute Malaysia, 52109 Kepong, Selangor, Malaysia.

[10]Department of Natural History, Royal Ontario Museum, Toronto, Canada

[11]School of Biological and Chemical Sciences, Queen Mary University of London, Mile End Road, London E1 4NS, United Kingdom

[12]NTNU University Museum, Norwegian University of Science and Technology, Trondheim, Norway

Abstract

Background:

Southeast (SE) Asia is one of the most biodiverse regions in the world and it holds approximately 20% of all mammal species. Despite this, the majority of South East (SE) Asia's genetic diversity is still poorly characterised. The growing interest in using environmental DNA (eDNA) to assess and monitor SE Asian species, in particular threatened mammals - has created the urgent need to expand the available reference database of mitochondrion barcode and complete mitogenome sequences. We have contributed to this need, by generating 75 new mitogenome sequences reconstructed from DNA isolated from a range of historical and modern tissue samples.

Findings:

Approximately 56 gigabases of raw sequence were generated. From this data we assembled 75 complete mitogenome sequences, with an average depth of coverage of 102.9x and 55.2x for modern samples and historical samples, respectively. This dataset represents 55 species, of which 32 species have currently no mitogenome available. The mitogenomes were geotagged to their sampling location, where known, to display a clearer geographical distribution of the species.

Conclusion:

Our new database of 55 taxa will greatly enhance the utility of eDNA approaches for monitoring mammals in SE Asia, as it greatly increases the likelihoods that identification of metabarcoding sequencing reads can be assigned to reference sequences. This magnifies the confidence in species detections and thus allows more robust surveys and monitoring programmes of SE Asia's threatened mammal biodiversity. The extensive collections of historical samples from SE Asia in western and SE Asian museums should serve as additional valuable material to further enrich this reference database.

Keywords:

invertebrate-derived (iDNA), metabarcoding, Malaysia, GenBank, Taxonomic assignment

Data description:

Context

Southeast (SE) Asia is one of the most biodiverse regions in the world, hosting ~20% of mammal species, but is experiencing rapid deforestation for agriculture and development. To assess the ecological consequences of land-use change, there is growing interest in using environmental DNA (eDNA) to monitor mammal populations, particularly threatened taxa that often underpin conservation policies. Yet current efforts are hampered by the lack of a reference database of mitochondrion barcodes and complete mitogenome sequences. We have contributed to this need, by generating 75 new mitogenome sequences reconstructed from DNA isolated from a range of historical and modern tissue samples.

DNA extraction

Genomic DNA was extracted from different sample types of 75 small mammals, comprising 55 species, listed in Table 1 and Table 2. DNA from modern tissue and blood samples was isolated using the Qiagen DNeasy extraction kit (Qiagen, Hilden, Germany) or Invitek DNA extraction kit (Invitek GmbH, Berlin, Germany), as per standard protocols. Historical samples obtained from the Zoological Museum, Natural History Museum of Denmark, University of Copenhagen (ZM, KU) were treated differently according to type of tissue (Additional file 1a), while at the German Primate Center DNA extraction from museum specimens followed Liedigk et al. (2015) [1]. Complete details of sample information are provided in Additional file 2.

Mitogenome sequencing, assembly and annotation

Mitogenomes were generated using several approaches. In Copenhagen, author FMS constructed Illumina shotgun libraries with insert sizes ranging between 50 bp to 400 bp. To construct libraries, DNA was sheared to the target size range using Bioruptor® XL (Diagenode, USA) and converted into an Illumina-compatible sequencing library using the NEBNext E6070 kit (New England Biolabs, UK). The libraries were PCR amplified with index primers and purified using Qiaquick columns (Qiagen, Hilden, Germany) according to the manufacturer's instruction (Additional file 1b). Multiple libraries were combined together into three pools, normalized to 10 nM and sequenced across three lanes of Illumina HiSeq 2500

using SR100bp chemistry. In Berlin and Goettingen, mitogenomes were generated by author PRP and CR using overlapping PCR products using long range PCR (Additional file 1c) followed by library construction and MiSeq sequencing, or Sanger sequencing as described in [2] and [1, 3, 4], respectively. Author RM's mitogenomes were done using methods outlined in [5].

Raw reads for FMS samples were assembled independently by authors FMS and FP using two different approaches, then compared for consistency. Author FMS trimmed the reads for sequencing adapters, low quality stretches and leading/tailing Ns using AdapterRemoval 1.2 [6]. The mitochondrial genome was reconstructed with MITObim v1.8 [7] using the reference mitogenome of the closest species available in GenBank as the seed reference (Additional file 2). In order to obtain the mapping statistics of the samples, we ran PALEOMIX v 1.2.6 [8] with default parameters where reads shorter than 25 bp after trimming were discarded. The trimmed reads were aligned against the newly assembled mitogenome generated by MITObim using Burrows–Wheeler Aligner (BWA) [9]. Alignments showing low-quality scores and PCR duplicates were further removed using the MarkDuplicates program from Picard tools, and reads were locally realigned around small insertions and deletions (indels) to improve overall genome quality using the IndelRealigner tool from the Genome Analysis Toolkit (GATK)[10]. In contrast, author FP inputted the trimmed reads into mitoMaker (https://sourceforge.net/projects/mitomaker/), which performs a de-novo and reference-based assembly using SOAPdenovoTrans v1.03 [11] and MITObim v1.7 [7]. Post assembly, the FMS and FP mitogenomes were manually compared for consistency by FMS to generate the final consensus sequences. These assemblies were automatically annotated using tRNAscan-SE v1.4 [12] and BLAST v2.2.29 [13] using the mitochondrial genomes found in the NCBI RefSeq (https://www.ncbi.nlm.nih.gov/refseq/) as references.

For mitogenome constructed by author RM, Illumina sequence reads were de-multiplexed according to the respective indexes with the Illumina software bcl2fastq v2.17 (Illumina, San Diego, CA, USA) and adapters were clipped from the sequence reads with the software cutadapt v1.3 [14]. Quality trimming was done through a sliding window approach (10 bp; Q20) and all reads shorter than 20 bp were removed from the analyses. Mitogenome references from target or closely related species were used for

mapping of the sequencing reads. Aligned reads were de-duplicated using MarkDuplicates from Picard-tools v1.106 (https://github.com/broadinstitute/picard). VariantCalling was carried out using Samtools v1.1 [9] and Bcftools v1.2 (http://github.com/samtools/bcftools). For each sample, GATK [10] Variant Calling output files were further filtered to have a minimum read coverage ≥ 3x, and variants were only called when the corresponding base was represented by ≥ 50%, otherwise this position was "N"-masked.

Numbers of raw reads generated for each samples and mapping statistics for all 75 mitogenome assemblies are shown in Additional file 2. Sanger sequenced mitogenomes were checked with 4Peaks 1.8 (www.nucleobytes.com), assembled with SeaView 4.5.4 [15] and annotated with DOGMA [16]. All mitogenomes were checked manually by eye to identify possible errors caused by insertion and deletions. The final mitochondrial genomes have been uploaded to GenBank (accession numbers are provided in Table 1 and Table 2). The details of all new mitogenomes assembled in this work are given in Table 1 and Table 2. Mitogenomes (62 samples) with known localities were geotagged and mapped to display its geographical distribution (Figure 1).

Phylogenetic analysis

All the sequenced mitogenomes were aligned using MAFFT v7.158b [17], using the E-INS-i option (Additional file 3). RAxML v8.0.26 [18] was used to perform the phylogenetic analysis with a GTR+GAMMA model of nucleotide substitution. To obtain node support, we used 100 bootstrap pseudo-replicates (Figure 2). The newick file is provided as Additional file 4.

Availability of supporting data

Raw shotgun data are deposited in the SRA under bioproject number PRJNA361218 and is available in the GigaScience repository, GigaDB ftp://user27:SallehFaezah@climb.genomics.cn.

Abbreviations

SE: South East; eDNA: environmental deoxyribonucleic acid; bp: basepair; PCR: polymerase chain reaction; BWA: Burrows–Wheeler Aligner; GATK: Genome Analysis Toolkit; BLAST: Basic Local Alignment Search Tool; NCBI RefSeq: National Center for Biotechnology Information Reference Sequence Database; MAFFT: Multiple Alignment using Fast Fourier Transform ; RAxML: Randomized Axelerated Maximum Likelihood.

Acknowledgements

This project was funded by the Malaysian Government, Lundbeck Foundation (R52-5062), Leibniz-Association grant SAW-2013-IZW-2, the German Federal Ministry of Education and Research grant BMBF FKZ: 01LN1301A and the German Primate Center. We thank the Danish National High-throughput Sequencing Centre for assistance in generating Illumina data. We thank the staff of the zoos in Hannover, Copenhagen, Gettorf, Belfast, Bristol, Howletts Wild Animal Park, the staff of the Ludwig-Maximilans-University Munich, the Bavarian State Collection Munich, the National Museums Scotland, Edinburgh, the Natural History Museum Berlin, Naturalis Leiden, and the Zoological Museum, Natural History Museum of Denmark, University of Copenhagen for providing samples for this work.

Authors' contributions

FMS, AW, and MTPG conceived the project. FMS, MHSS, MSS, MSA, RM, PRP, CR, BKL, and SJR collected the samples and extracted the genomic DNA. FMS, RM, PRP and CR constructed the libraries and did sequencing. FMS, JRM, FP, SL, PRP, RM, DL and CR assembled the mitogenomes and performed mitogenome analysis. FMS, SL, PRP and MTPG wrote the article. All authors discussed the project and data. All authors read and approved the final manuscript.

Competing interests

The authors declare that they have no competing interests.

Author details

[1]Centre for GeoGenetics, Natural History Museum of Denmark, University of Copenhagen, Øster Voldgade 5-7, 1350, Copenhagen, Denmark. [2]Faculty of Biosciences and Medical Engineering, Universiti Teknologi Malaysia, 81310 Johor Bahru, Johor, Malaysia. [3]Leibniz Institute for Zoo and Wildlife Research, Alfred-Kowalke Strasse 17, 10315 Berlin, Germany. [4]Undergraduate Program on Genomic Sciences, Universidad Nacional Autonoma de Mexico, 62210 Cuernavaca, Mexico. [5]BGI-Shenzhen, Shenzhen, GuangDong, China. [6]Natural History Museum, University of Oslo, P.O. Box 1172 Blindern, NO-0318, Oslo, Norway. [7]Freie Universität Berlin, Kaiserswerther Str. 16-18, 14195 Berlin, Germany. [8]Gene Bank of Primates and Primate Genetics Laboratory, German Primate Center, Leibniz Institute for Primate Research, Kellnerweg 4, 37077, Göttingen, Germany. [9]Forest Biodiversity Division, Forest Research Institute Malaysia, 52109 Kepong, Selangor, Malaysia. [10]Department of Natural History, Royal Ontario Museum, Toronto, Canada. [11]School of Biological and Chemical Sciences, Queen Mary University of London, Mile End Road, London E1 4NS, UK. [12]NTNU University Museum, Norwegian University of Science and Technology, Trondheim, Norway.

References

1. Liedigk R, Kolleck J, Böker KO, et al (2015) Mitogenomic phylogeny of the common long-tailed macaque (Macaca fascicularis fascicularis). BMC Genomics 16:222
2. Patel RP, Förster DW, Kitchener AC, et al (2016) Two species of Southeast Asian cats in the genus Catopuma with diverging histories: an island endemic forest specialist and a widespread habitat generalist. Open Science 3:160350
3. Roos C, Zinner D, Kubatko LS, et al (2011) Nuclear versus mitochondrial DNA: evidence for hybridization in colobine monkeys. BMC Evol Biol 11:77
4. Liedigk R, Roos C, Brameier M, Zinner D (2014) Mitogenomics of the Old World monkey tribe Papionini. BMC Evol Biol 14:176
5. Fortes GG, Paijmans JLA (2015) Analysis of Whole Mitogenomes from Ancient Samples. Methods Mol Biol 1347:179–195
6. Lindgreen S (2012) AdapterRemoval: easy cleaning of next-generation sequencing reads. BMC Res Notes 5:337
7. Hahn C, Bachmann L, Chevreux B (2013) Reconstructing mitochondrial genomes directly from genomic next-generation sequencing reads—a baiting and iterative mapping approach. Nucleic Acids Res.
8. Schubert M, Ermini L, Der Sarkissian C, et al (2014) Characterization of ancient and modern genomes by SNP detection and phylogenomic and metagenomic analysis using PALEOMIX. Nat Protoc 9:1056–1082
9. Li H, Durbin R (2009) Fast and accurate short read alignment with Burrows–Wheeler transform. Bioinformatics 25:1754–1760
10. McKenna A, Hanna M, Banks E, et al (2010) The Genome Analysis Toolkit: a MapReduce framework for analyzing next-generation DNA sequencing data. Genome Res 20:1297–1303
11. Xie Y, Wu G, Tang J, et al (2014) SOAPdenovo-Trans: de novo transcriptome assembly with short RNA-Seq reads. Bioinformatics 30:1660–1666
12. Lowe TM, Eddy SR (1997) tRNAscan-SE: a program for improved detection of transfer RNA genes in genomic sequence. Nucleic Acids Res 25:955–964
13. Camacho C, Coulouris G, Avagyan V, Ma N, Papadopoulos J, Bealer K, Madden TL (2009) BLAST+: architecture and applications. BMC Bioinformatics 10:421
14. Martin M (2011) Cutadapt removes adapter sequences from high-throughput sequencing reads. EMBnet.journal 17:10–12
15. Gouy M, Guindon S, Gascuel O (2010) SeaView version 4: A multiplatform graphical user interface

for sequence alignment and phylogenetic tree building. Mol Biol Evol 27:221–224

16. Wyman SK, Jansen RK, Boore JL (2004) Automatic annotation of organellar genomes with DOGMA. Bioinformatics 20:3252–3255

17. Katoh K, Standley DM (2013) MAFFT multiple sequence alignment software version 7: improvements in performance and usability. Mol Biol Evol 30:772–780

18. Stamatakis A (2014) RAxML version 8: a tool for phylogenetic analysis and post-analysis of large phylogenies. Bioinformatics 30:1312–1313

Tables and Figures

1. Table 1: List of mitogenomes assembled in this work that supplement pre-existing mitogenome references already available in GenBank
2. Table 2: List of mitogenomes assembled in this work that currently have no complete mitogenome reference available in GenBank
3. Figure 1: Geographical distribution of mitogenomes assembled in this work (62 mitogenomes with known locality)
4. Figure 2: Phylogenetic tree of mitogenomes assembled in this work

Additional files

1. Additional file 1: DNA extraction of historical samples, library construction and primer information
2. Additional file 2: Sample information sheet of mitogenomes assembled in this work
3. Additional file 3: Alignment of mitogenomes assembled in this work
4. Additional file 4: Newick file for phylogenetic tree of mitogenomes assembled in this work

 Click here to download Table Table 1_Salleh_etal.xlsx

Table 1: List of mitogenomes assembled in this work that supplement pre-existing mitogenome references already available in GenBank

No	GenBank ID	Common name	Genus	Species	Assembly Size	Locality	Source	Sample date of collection	Data by
1	KY117537	*Hog deer*	*Axis*	*porcinus*	16402	CPH Zoo	ZM, KU	21/8/1912	FMS/FP
2	KY117538	*Pallas's squirrel*	*Callosciurus*	*erythraeus*	16656	Bangkok, Thailand	ZM, KU	25/5/1969	FMS/FP
3	KX265095	*Bay Cat*	*Catopuma*	*badia*	16,960	Sabah, Malaysia	National Museums Scotland	20/04/2000	PRP
4	KX224524	*Asiatic Golden Cat*	*Catopuma*	*temminckii*	16,960	Thailand	American Museum of National History, New York.	10/10/1927	PRP
5	KY117545	*Sumatran Rhino*	*Dicerorhinus*	*sumatrensis*	16466	Sumatra	Naturalis, Leiden, The Netherlands	1880	RM
6	KY117546	*Least pygmy squirrel*	*Exilisciurus*	*exilis*	16637	Indonesia	ROM	16/06/1993	FMS/FP
7	KY117548	*Hose's mongoose*	*Herpestes*	*javanicus*	16340	Java, Indonesia	ZM, KU	12/3/1947	FMS/FP
8	KY117550	*Three-striped ground Squirrel*	*Lariscus*	*indsignis*	16399	Maybe Malaysia	ZM, KU	unknown	FMS/FP
9	KY117592	*Black Crested Macaque*	*Macaca*	*nigra*	16558	captive	Gettorf Zoo, Germany	18/07/2000	CR
10	KY117593	*Northern Pig-tailed Macaque*	*Macaca*	*leonina*	16554	captive	Ludwig-Maximilans-University Munich, Germany	6/3/1995	CR
11	KY117594	*Southern pig-tailed macaque*	*Macaca*	*nemestrina*	16531	West Malaysia	National Museums Scotland, Edinburgh, UK	unknown	CR
12	KY117561	*Reeves's/Chinese Muntjak*	*Muntiacus*	*reevesi*	16354	Shin-Yi, Taiwan	Naturalis, Leiden, The Netherlands	1968	RM
13	KT288227	*Marbled Cat*	*Pardofelis*	*marmorata*	17,218	Sumatra	National archaeological museum of the Netherlands, Leiden	30/08/1930	PRP
14	KY117602	*Sumatra Surili*	*Presbytis*	*melalophos*	16,558	captive	Howletts Wild Animal Park, UK	23/7/1999	CR
15	KR135743	*Flat-headed Cat*	*Prionailurus*	*planiceps*	17,074	Sabah, Malaysia	Sabah wildlife department	25/04/2000	PRP
16	KY117580	*Malayan Field Rat*	*Rattus*	*tiomanicus*	16415	SPF Bidor, Perak, Malaysia	FRIM	12/2/2011	FMS/FP
17	KY117579	*Malayan Field Rat*	*Rattus*	*tiomanicus*	16312	Indonesia	ROM	01/06/1993	FMS/FP

18	KY117581	*Malayan Field Rat*	*Rattus*	*tiomanicus*	16305	Hutan Simpan Chikus, Tapah Perak, Malaysia	FRIM	13/1/2011	FMS/FP
19	KY117582	*Black Giant Squirrel*	*Ratufa*	*bicolor peninsulae*	16600	Bang Nara, Malakka, Thailand	ZM, KU	3/12/1932	FMS/FP
20	KY117574	*Javan Rhino*	*Rhinoceros*	*sondaicus*	16417	Java	Copenhagen Natural History Museum	unknown	RM
21	KY117575	*Javan Rusa*	*Rusa*	*timorensis*	16437	Toeloeng Agoeng, West Java	Naturalis, Leiden, The Netherlands	unknown	RM
22	KY117576	*Indian Sambar Deer*	*Rusa*	*unicolor dejeani*	16437	Mentawai, Indonesia	Naturalis, Leiden, The Netherlands	unknown	RM
23	KY117599	*Western Purple-faced Langur*	*Semnopithecu s*	*vetulus*	16545	captive	Belfast Zoo, UK	9/11/1998	CR
24	KY117589	*Malayan Tapir*	*Tapirus*	*indicus*	16794	captive	Copenhagen Zoo	11/1/2015	FMS/FP
25	KY117598	*Silvered Langur*	*Trachypithecu s*	*cristatus*	16551	North Sumatra	Bavarian State Collection Munich, Germany	1911	CR

ZM, KU: Zoological Musuem, University of Copenhagen; ROM: Royal Ontario Museum; FRIM: Forest Research Institute, Malaysia

Table 2: List of mitogenomes assembled in this work that currently have no complete mitogenome reference available in GenBank

No	GenBank ID	Common name	Genus	Species	Assembly Size	Locality	Source	Sample date of collection	Data by
1	KY117536	*Asian Small-clawed Otter*	*Aonyx*	*cinereus*	16153	captive	Copenhagen Zoo	08/08/11	FMS/FP
2	KY117535	*Asian Small-clawed Otter*	*Aonyx*	*cinereus*	16153	Sarawak, Malaysia	British Musuem of Natural History, London	25/8/2010	FMS/FP
3	KY117560	*Binturong*	*Arctictis*	*binturong*	17,067	unknown	Tierpark, Berlin	29/11/2010	PRP
4	KY117541	*Plantain Squirrel*	*Callosciurus*	*notatus*	16582	Hutan Bidor, Perak, Malaysia	FRIM	11/2/2011	FMS/FP
5	KY117542	*Plantain squirrel*	*Callosciurus*	*notatus*	16599	East Kalimantan, Indonesia	ROM	03/06/1993	FMS/FP
6	KY117543	*Prevost's squirrel*	*Callosciurus*	*prevostii*	16674	East Kalimantan, Indonesia	ROM	15/06/1993	FMS/FP
7	KY117540	*Variable squirrel*	*Callosciurus*	*finlaysonii frandseni*	15747	Koh Chang	ZM, KU	14/1/1900	FMS/FP
8	KY117539	*Variable squirrel*	*Callosciurus*	*finlaysonii*	16489	Central Siam	ZM, KU	2/2/1928	FMS/FP
9	KY117544	*Sunda Otter Civet*	*Cynogale*	*bennetti*	15784	Borneo	British Musuem of Natural History, London	25/8/2010	FMS/FP
10	KY117547	*Hose's mongoose*	*Herpestes*	*hosei*	16819	Ceylon	ZM, KU	1/7/1931	FMS/FP
11	KY117549	*Thick-spined porcupine*	*Hystrix*	*crassipinis*	15778	CPH Zoo	ZM, KU	19/1/1874	FMS/FP
12	KY117552	*Long-tailed Giant Rat*	*Leopaldamys*	*sabanus*	15973	G. Telapak Buruk, Negeri Sembilan, Malaysia	FRIM	24/2/2010	FMS/FP
13	KY117553	*Long-tailed Giant Rat*	*Leopaldamys*	*sabanus*	15972	Teluk Segadas, P. Pangkor, Perak, Malaysia	FRIM	19/3/2010	FMS/FP
14	KY117554	*Long-tailed Giant Rat*	*Leopaldamys*	*sabanus*	15974	Hutan Simpan Temengor,Gerik Perak, Malaysia	FRIM	23/1/2014	FMS/FP
15	KY117555	*Long-tailed Giant Rat*	*Leopaldamys*	*sabanus*	15972	Hutan Simpan Lenggor, Kluang, Johor, Malaysia	FRIM	19/2/2014	FMS/FP
16	KY117551	*Long-tailed giant rat*	*Leopaldamys*	*sabanus*	15974	Malaysia	ROM	28/05/1993	FMS/FP
17	KY117556	*Hairy-nosed otter*	*Lutra*	*sumatrana*	16580	Bang Nara, Thailand	ZM, KU	1/4/1939	FMS/FP

18	KY117557	*Smooth-coated otter*	*Lutrogale*	*perspicillata*	16042	Malacca, Malaysia	British Musuem of Natural History, London	25/8/2010	FMS/FP
19	KY117558	*Smooth-coated otter*	*Lutrogale*	*perspicillata*	16041	Bang Nara, Thailand	ZM, KU	24/1/1933	FMS/FP
20	KY117591	*Moor macaque*	*Macaca*	*maura*	16563	captive	Hannover Zoo, Germany	20/8/1998	CR
21	KY117564	*Rajah/Brown Spiny Rat*	*Maxomys*	*rajah*	16200	Indonesia	ROM	06/06/1993	FMS/FP
22	KY117562	*Rajah/Brown Spiny Rat*	*Maxomys*	*rajah*	16296	Teluk Segadas, P. Pangkor, Perak, Malaysia	FRIM	19/3/2010	FMS/FP
23	KY117563	*Rajah/Brown Spiny Rat*	*Maxomys*	*rajah*	16296	Pasir Bogak, P.Pangkor, Perak, Malaysia	FRIM	18/3/2010	FMS/FP
24	KY117567	*Red Spiny Rat*	*Maxomys*	*surifer*	16286	50 ha, Pasoh, Negeri Sembilan, Malaysia	FRIM	12/6/2008	FMS/FP
25	KY117566	*Red Spiny Rat*	*Maxomys*	*surifer*	16290	Indonesia	ROM	21/05/1993	FMS/FP
26	KY117565	*Red Spiny Rat*	*Maxomys*	*surifer*	16286	Malaysia	ROM	17/05/2013	FMS/FP
27	KY117570	*Whitehead's Spiny Rat*	*Maxomys*	*whiteheadi*	16316	Hutan Simpan Bikam, Perak, Malaysia	FRIM	12/2/2011	FMS/FP
28	KY117571	*Whitehead's Spiny Rat*	*Maxomys*	*whiteheadi*	16316	Keruing Trail, FRIM, Kepong, Selangor, Malaysia	FRIM	13/3/2013	FMS/FP
29	KY117568	*Whitehead's Spiny Rat*	*Maxomys*	*whiteheadi*	16287	Hutan Simpan Bikam, Perak, Malaysia	FRIM	12/2/2011	FMS/FP
30	KY117569	*Whitehead's Spiny Rat*	*Maxomys*	*whiteheadi*	16429	Bukit Tapah, Perak, Malaysia	FRIM	23/3/2011	FMS/FP
31	KY052142	*Indian muntjac*	*Muntiacus*	*muntjak*	16354	West Java	Vienna NHM	1858	RM
32	KY117559	*Bornean yellow muntjac*	*Muntiacus*	*atherodes*	16354	Koemai, West Borneo	Bonn NHM	1938	RM
33	KY052092	*Indian Red Muntjac*	*Muntiacus*	*vaginalis*	16354	Gangtok, South Sikkim, India	Berlin NHM	unknown	RM
34	KY117573	*Dark-tailed Tree Rat*	*Niviventer*	*cremoriventer*	16322	Track 5 (G.Inas), Kedah, Malaysia	FRIM	5/11/2009	FMS/FP
35	KY117572	*Dark-tailed tree rat*	*Niviventer*	*cremoriventer*	16234	Malaysia	ROM	17/05/2013	FMS/FP

36	KY117600	*Grizzled Leaf Monkey*	*Presbytis*	*comata comata*	16551	captive	Howletts Wild Animal Park, UK	23/12/1999	CR
37	KY117601	*Mitred Leaf Monkey*	*Presbytis*	*mitrata*	16,555	captive	Howletts Wild Animal Park, UK	12/11/1998	CR
38	KX857784	*Leopard cat*	*Prionailurus*	*bengalensis*	16,989	Thailand	American Museum of National History, New York.	25/02/1924	PRP
39	KY117578	*Annandale’s Sundaic Rat*	*Rattus*	*annandalei*	16297	Hutan Simpan Bikam, Perak, Malaysia	FRIM	12/2/2011	FMS/FP
40	KY117577	*Annandale’s Sundaic Rat*	*Rattus*	*annandalei*	16301	Hutan Simpan Bikam, Perak, Malaysia	FRIM	11/2/2011	FMS/FP
41	KY117583	*Mountain giant Sunda rat*	*Sundamys*	*infraluteus*	16297	Malaysia	ROM	18/05/2013	FMS/FP
42	KY117585	*Müller's Giant Sunda Rat*	*Sundamys*	*meulleri*	16326	Track 1 (G.Inas), Kedah, Malaysia	FRIM	5/11/2009	FMS/FP
43	KY117584	*Müller's giant Sunda rat*	*Sundamys*	*meulleri*	16304	Malaysia	ROM	01/06/2013	FMS/FP
44	KY117586	*Brooke's squirrel*	*Sundasciurus*	*brookei*	16417	East Kalimantan, Indonesia	ROM	13/06/1993	FMS/FP
45	KY117587	*Low's squirrel*	*Sundasciurus*	*lowii*	16307	East Kalimantan, Indonesia	ROM	06/06/1993	FMS/FP
46	KY117588	*name pending*	*Sundasciurus*	*sp*	16458	East Kalimantan, Indonesia	ROM	21/06/1993	FMS/FP
47	KY117595	*Phayre's Langur*	*Trachypithecus*	*phayrei phayrei*	16548	South West Myanmar	Natural History Museum Berlin, Germany	unknown	CR
48	KY117596	*East Javan Ebony Langur*	*Trachypithecus*	*auratus*	16552	captive	Bristol Zoo, UK	26/10/2010	CR
49	KY117597	*West Javan Ebony Langur*	*Trachypithecus*	*mauritius*	16554	West Java	Naturalis Leiden; Netherlands	unknown	CR
50	KY117590	*Long-tailed porcupine*	*Trichys*	*fasciculata*	16328	Borneo	ZM, KU	5/10/1894	FMS/FP

ZM, KU: Zoological Musuem, University of Copenhagen; ROM: Royal Ontario Museum; FRIM: Forest Research Institute, Malaysia; NHM: Natural History

' Musuem

Figure 1 Geographical distribution of mitogenomes assembled in this work

Click here to download Figure Figure 1_Sallehetal.pdf

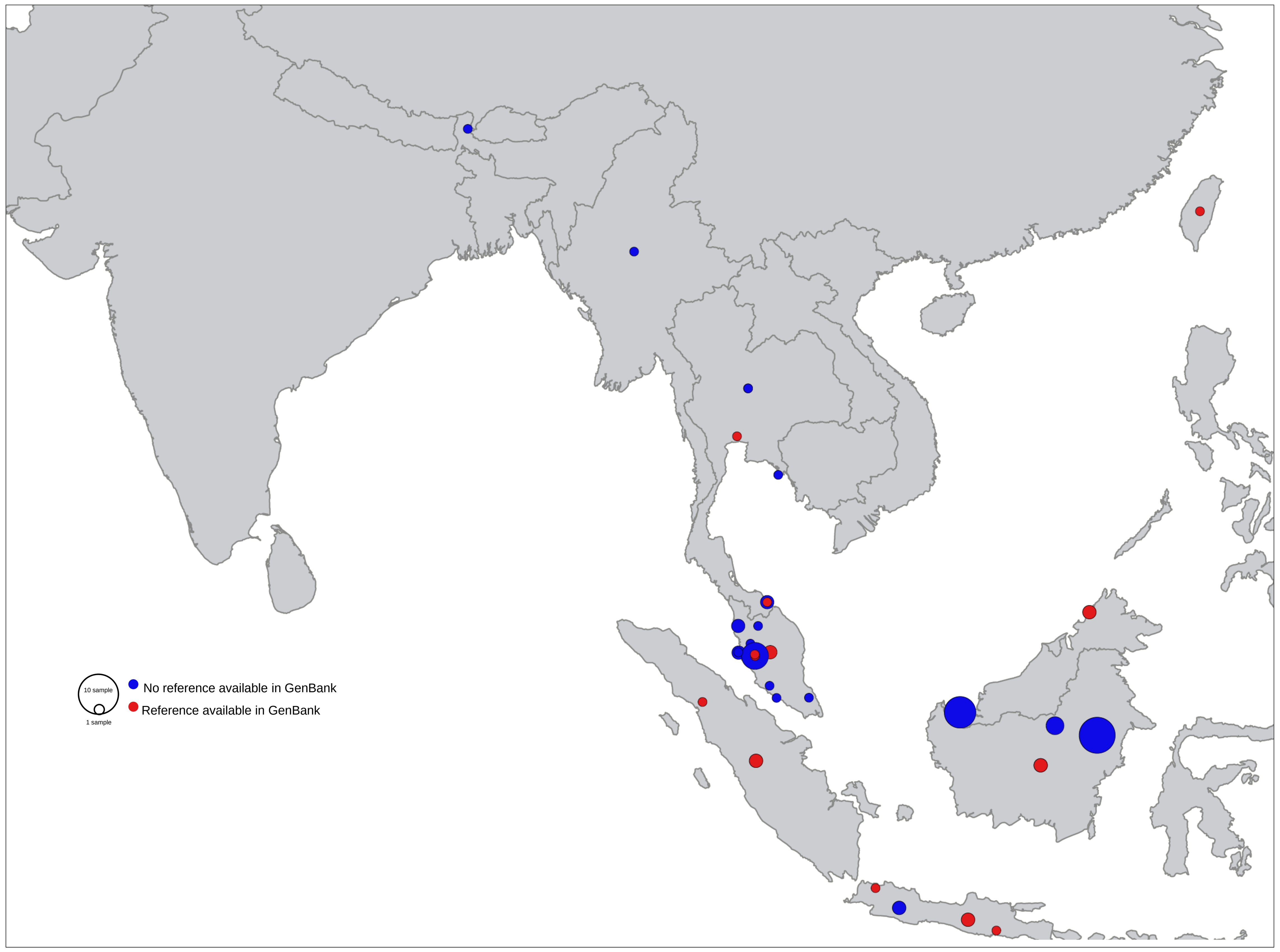

Figure 2 Phylomitogenetic tree of mitogenomes assembled in this work

Click here to download Figure Figure 2_Sallehetal.pdf

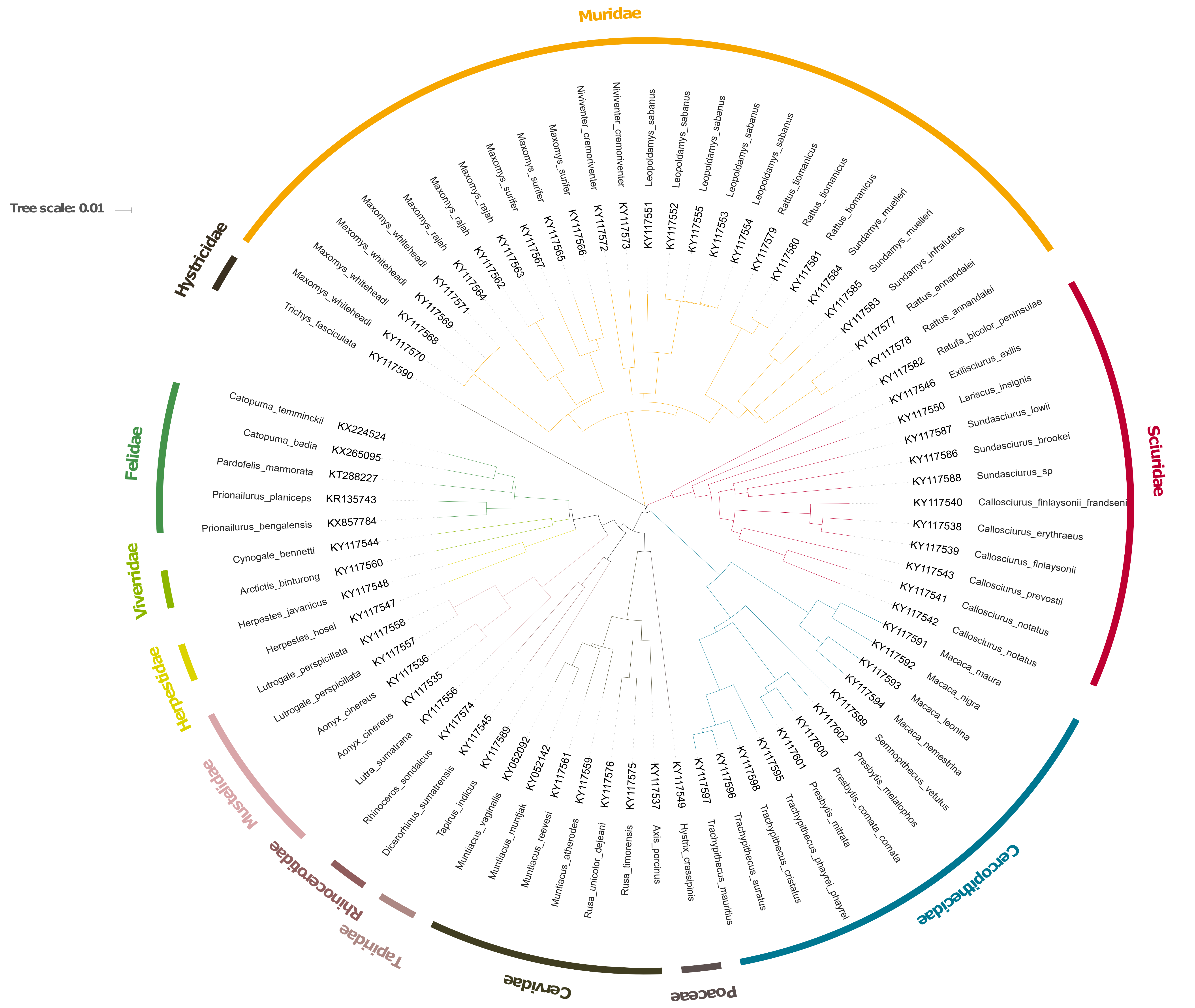

Click here to access/download
Supplementary Material
1 Data information_Sallehetal.docx

Click here to access/download
Supplementary Material
1 README file_Salleh.txt

Additional file 1 DNA extraction for historical samples and primer information

Click here to access/download
Supplementary Material
Additional file 1_Sallehetal.docx

Additional file 2: Sample information sheet of 75 SE asian small mammal mitogenomes

Click here to access/download
Supplementary Material
Additional file 2_Sallehetal.xlsx

Additional file 3 Alignment of mitogenomes assembled in this work

Click here to access/download
Supplementary Material
Additional file 3_Sallehetal.fa

Additional file 4: newick

Click here to access/download
Supplementary Material
Additional file 4 newick.txt
