# Supplementary material for: An expanded mammal mitogenome dataset from Southeast Asia
Source: Gigascience. 2017 Jul 13;6(8):1–8. doi: 10.1093/gigascience/gix053 (PMC5737531; doi:10.1093/gigascience/gix053)
Supplement: GIGA-D-17-00015_Revision-1.pdf [file gix053_giga-d-17-00015_revision-1.pdf]

Manuscript main text salleh et al
Click here to download Manuscript 1ManuscriptmaintextSallehetal_revised.docx

Title: An expanded mammal mitogenome dataset from Southeast Asia

Faezah Mohd Salleh[1,2*], Jazmín Ramos-Madrigal[1], Fernando Peñaloza[3,4], Shanlin Liu[1,5], Mikkel-Holger S. Sinding[1,6], Riddhi P. Patel[3,7], Renata Martins[3], Dorina Lenz[3] Jörns Fickel[3,8], Christian Roos[9], Mohd Shahir Shamsir[2], Mohammad Shahfiz Azman[10], Burton K. Lim[11], Stephen J. Rossiter[12], Andreas Wilting[3], M. Thomas P. Gilbert[1,13*]

*Correspondence: faezah@fbb.utm.my and tgilbert@snm.ku.dk

[1]Centre for GeoGenetics, Natural History Museum of Denmark, University of Copenhagen, Øster Voldgade 5-7, 1350, Copenhagen, Denmark

[2]Faculty of Biosciences and Medical Engineering, Universiti Teknologi Malaysia, 81310 Johor Bahru, Johor, Malaysia

[3]Leibniz Institute for Zoo and Wildlife Research, Alfred-Kowalke Strasse 17, 10315 Berlin, Germany.

[4]Undergraduate Program on Genomic Sciences, Universidad Nacional Autonoma de Mexico, 62210 Cuernavaca, Mexico

[5]BGI-Shenzhen, Shenzhen, GuangDong, China

[6]Natural History Museum, University of Oslo, P.O. Box 1172 Blindern, NO-0318, Oslo, Norway

[7]Freie Universität Berlin, Kaiserswerther Str. 16-18, 14195 Berlin, Germany

[8]University of Potsdam, Institute for Biochemistry and Biology, Karl-Liebknecht-Str 24-25, 14476 Potsdam, Germany

[9]Gene Bank of Primates and Primate Genetics Laboratory, German Primate Center, Leibniz Institute for Primate Research, Kellnerweg 4, 37077, Göttingen, Germany

[10]Forest Biodiversity Division, Forest Research Institute Malaysia, 52109 Kepong, Selangor, Malaysia.

[11]Department of Natural History, Royal Ontario Museum, Toronto, Canada

[12]School of Biological and Chemical Sciences, Queen Mary University of London, Mile End Road, London E1 4NS, United Kingdom

[13]NTNU University Museum, Norwegian University of Science and Technology, Trondheim, Norway

Abstract

Background:

Southeast (SE) Asia is one of the most biodiverse regions in the world and it holds approximately 20% of all mammal species. Despite this, the majority of SE Asia's genetic diversity is still poorly characterised. The growing interest in using environmental DNA (eDNA) to assess and monitor SE Asian species, in particular threatened mammals - has created the urgent need to expand the available reference database of mitochondrial barcode and complete mitogenome sequences. We have partially addressed this need by generating 72 new mitogenome sequences reconstructed from DNA isolated from a range of historical and modern tissue samples.

Findings:

Approximately 55 gigabases of raw sequence were generated. From this data we assembled 72 complete mitogenome sequences, with an average depth of coverage of 102.9x and 55.2x for modern samples and historical samples, respectively. This dataset represents 52 species, of which 30 species had no previous mitogenome data available. The mitogenomes were geotagged to their sampling location, where known, to display a detailed geographical distribution of the species.

Conclusion:

Our new database of 52 taxa will strongly enhance the utility of eDNA approaches for monitoring mammals in SE Asia, as it greatly increases the likelihoods that identification of metabarcoding sequencing reads can be assigned to reference sequences. This magnifies the confidence in species detections and thus allows more robust surveys and monitoring programmes of SE Asia's threatened mammal biodiversity. The extensive collections of historical samples from SE Asia in western and SE Asian museums should serve as additional valuable material to further enrich this reference database.

Keywords:

invertebrate-derived (iDNA), metabarcoding, GenBank, Taxonomic assignment

Data description:

Context

Southeast (SE) Asia is one of the most biodiverse regions in the world, hosting ~20% of mammal species, but is experiencing rapid deforestation for agriculture and development. To assess the ecological consequences of land-use change, there is growing interest in using environmental DNA (eDNA) to monitor mammal populations, particularly threatened taxa that often underpin conservation policies [1–4]. Yet current efforts are hampered by the lack of a reference database of mitochondrial barcodes and complete mitogenome sequences. Currently there are 922 mammalian mitogenomes available in Genbank. Unfortunately most are not tagged by location/origin. Data mining through manual screening of each mitogenomes resulted in 174 terrestrial mammal species which are typical to SE Asia. In this work, 30 novel species are added, contributing to ~17% expansion of the current SE Asia mammal mitogenome database.

DNA extraction

Genomic DNA was extracted from different sample types of 72 small mammals, comprising 52 species, listed in Table 1 and Table 2. DNA from modern tissue and blood samples was isolated using the Qiagen DNeasy extraction kit (Qiagen, Hilden, Germany) or Invitek DNA extraction kit (Invitek GmbH, Berlin, Germany), as per standard protocols following the manufacturer's guidelines. Historical samples obtained from the Zoological Museum, Natural History Museum of Denmark, University of Copenhagen (ZM, KU) were treated differently according to type of tissue (Additional file 1a), while at the German Primate Center DNA extraction from museum specimens followed Liedigk et al. (2015) [5]. Complete details of sample information are provided in Additional file 2.

Mitogenome sequencing, assembly and annotation

Mitogenomes were generated using several approaches. In Copenhagen, author FMS constructed Illumina shotgun libraries with insert sizes ranging between 50 bp to 400 bp. To construct libraries, DNA was sheared to the target size range using Bioruptor® XL (Diagenode, USA) and converted into an Illumina-compatible sequencing library using the NEBNext E6070 kit (New England Biolabs, UK). The

libraries were PCR amplified with index primers and purified using Qiaquick columns (Qiagen, Hilden, Germany) according to the manufacturer's instruction (Additional file 1b). Multiple libraries were combined together into three pools, normalized to 10 nM and sequenced across three lanes of Illumina HiSeq 2500 using SR100bp chemistry. In Berlin and Goettingen, mitogenomes were generated by author PRP and CR using overlapping PCR products using long range PCR (Additional file 1c) followed by library construction and MiSeq sequencing, or Sanger sequencing as described in [6] and [5, 7, 8], respectively. Author RM's mitogenomes were done using methods outlined in [9].

Raw reads for FMS samples were assembled independently by authors FMS and FP using two different approaches, then compared for consistency. Author FMS trimmed the reads for sequencing adapters, low quality stretches and leading/tailing Ns using AdapterRemoval 1.2 [10]. The mitochondrial genome was reconstructed with MITObim v1.8 [11] using the reference mitogenome of the closest species available in GenBank as the seed reference (Additional file 2). In order to obtain the mapping statistics of the samples, we ran PALEOMIX v 1.2.6 [12] with default parameters where reads shorter than 25 bp after trimming were discarded. The trimmed reads were aligned against the newly assembled mitogenome generated by MITObim using Burrows–Wheeler Aligner (BWA) [13]. Alignments showing low-quality scores and PCR duplicates were further removed using the MarkDuplicates program from Picard tools, and reads were locally realigned around small insertions and deletions (indels) to improve overall genome quality using the IndelRealigner tool from the Genome Analysis Toolkit (GATK)[14]. In contrast, author FP inputted the trimmed reads into mitoMaker (https://sourceforge.net/projects/mitomaker/), which performs a de-novo and reference-based assembly using SOAPdenovoTrans v1.03 [15] and MITObim v1.7 [11]. Post assembly, the FMS and FP mitogenomes were manually compared for consistency by FMS to generate the final consensus sequences. These assemblies were automatically annotated using tRNAscan-SE v1.4 [16] and BLAST v2.2.29 [17] using the mitochondrial genomes found in the NCBI RefSeq (https://www.ncbi.nlm.nih.gov/refseq/) as references.

For mitogenome constructed by author RM, Illumina sequence reads were de-multiplexed according to the respective indexes with the Illumina software bcl2fastq v2.17 (Illumina, San Diego, CA, USA) and

adapters were clipped from the sequence reads with the software cutadapt v1.3 [18]. Quality trimming was done through a sliding window approach (10 bp; Q20) and all reads shorter than 20 bp were removed from the analyses. Mitogenome references from target or closely related species were used for mapping of the sequencing reads. Aligned reads were de-duplicated using MarkDuplicates from Picard-tools v1.106 (https://github.com/broadinstitute/picard). VariantCalling was carried out using Samtools v1.1 [13] and Bcftools v1.2 (http://github.com/samtools/bcftools). For each sample, GATK [14] Variant Calling output files were further filtered to have a minimum read coverage ≥ 3x, and variants were only called when the corresponding base was represented by ≥ 50%, otherwise this position was "N"-masked.

Numbers of raw reads generated for each samples and mapping statistics for all 72 mitogenome assemblies are shown in Additional file 2. Sanger sequenced mitogenomes were checked with 4Peaks 1.8 (www.nucleobytes.com), assembled with SeaView 4.5.4 [19] and annotated with DOGMA [20]. All mitogenomes were checked manually by eye to identify possible errors caused by insertion and deletions in Tablet [21]. The final mitochondrial genomes have been uploaded to GenBank (accession numbers are provided in Table 1 and Table 2). The details of all new mitogenomes assembled in this work are given in Table 1 and Table 2. Mitogenomes (60 samples) with known localities were geotagged and mapped to display its geographical distribution (Figure 1).

Phylogenetic analysis

All the sequenced mitogenomes were aligned using MAFFT v7.158b [22], using the E-INS-i option (Additional file 3). RAxML v8.0.26 [23] was used to perform the phylogenetic analysis with a GTR+GAMMA model of nucleotide substitution. To obtain node support, we used 100 bootstrap pseudo-replicates (Figure 2). The newick file is provided as Additional file 4.

Availability of supporting data

Raw shotgun data are deposited in the SRA under bioproject number PRJNA361218 and is available in the GigaScience repository, GigaDB ftp://user27:SallehFaezah@climb.genomics.cn.

Abbreviations

SE: South East; eDNA: environmental deoxyribonucleic acid; bp: basepair; PCR: polymerase chain reaction; BWA: Burrows–Wheeler Aligner; GATK: Genome Analysis Toolkit; BLAST: Basic Local Alignment Search Tool; NCBI RefSeq: National Center for Biotechnology Information Reference Sequence Database; MAFFT: Multiple Alignment using Fast Fourier Transform ; RAxML: Randomized Axelerated Maximum Likelihood.

Acknowledgements

This project was funded by the Malaysian Government, Lundbeck Foundation (R52-5062), Leibniz-Association grant SAW-2013-IZW-2, the German Federal Ministry of Education and Research grant BMBF FKZ: 01LN1301A and the German Primate Center. We thank the Danish National High-throughput Sequencing Centre for assistance in generating Illumina data. We also thank the staff of the zoos in Hannover, Copenhagen, Gettorf, Belfast, Bristol, Howletts Wild Animal Park, the staff of the Ludwig-Maximilans-University Munich, the Bavarian State Collection Munich, the National Museums Scotland, Edinburgh, the Natural History Museum Berlin, Naturalis Leiden, and the Zoological Museum, Natural History Museum of Denmark, University of Copenhagen for providing samples for this work. BKL gratefully acknowledges the Indonesian and Malaysian government authorities for issuing scientific permits.

Authors' contributions

FMS, AW, JF and MTPG conceived the project. FMS, MHSS, MSS, MSA, RM, PRP, CR, BKL, and SJR collected the samples and extracted the genomic DNA. FMS, RM, PRP and CR constructed the libraries and did sequencing. FMS, JRM, FP, SL, PRP, RM, DL and CR assembled the mitogenomes and performed mitogenome analysis. FMS, SL, PRP and MTPG wrote the article. All authors discussed the project and data. All authors read and approved the final manuscript.

Competing interests

The authors declare that they have no competing interests.

Author details

[1]Centre for GeoGenetics, Natural History Museum of Denmark, University of Copenhagen, Øster Voldgade 5-7, 1350, Copenhagen, Denmark. [2]Faculty of Biosciences and Medical Engineering, Universiti Teknologi Malaysia, 81310 Johor Bahru, Johor, Malaysia. [3]Leibniz Institute for Zoo and Wildlife Research, Alfred-Kowalke Strasse 17, 10315 Berlin, Germany. [4]Undergraduate Program on Genomic Sciences, Universidad Nacional Autonoma de Mexico, 62210 Cuernavaca, Mexico. [5]BGI-Shenzhen, Shenzhen, GuangDong, China. [6]Natural History Museum, University of Oslo, P.O. Box 1172 Blindern, NO-0318, Oslo, Norway. [7]Freie Universität Berlin, Kaiserswerther Str. 16-18, 14195 Berlin, Germany. [8]University of Potsdam, Institute for Biochemistry and Biology, Karl-Liebknecht-Str 24-25, 14476 Potsdam, Germany. [9]Gene Bank of Primates and Primate Genetics Laboratory, German Primate Center, Leibniz Institute for Primate Research, Kellnerweg 4, 37077, Göttingen, Germany. [10]Forest Biodiversity Division, Forest Research Institute Malaysia, 52109 Kepong, Selangor, Malaysia. [11]Department of Natural History, Royal Ontario Museum, Toronto, Canada. [12]School of Biological and Chemical Sciences, Queen Mary University of London, Mile End Road, London E1 4NS, United Kingdom. [13]NTNU University Museum, Norwegian University of Science and Technology, Trondheim, Norway.

References

1. Bohmann K, Evans A, Gilbert MTP, Carvalho GR, Creer S, Knapp M, Yu DW, de Bruyn M (2014) Environmental DNA for wildlife biology and biodiversity monitoring. Trends Ecol Evol 29:358–367
2. Lee P-S, Gan HM, Clements GR, Wilson J-J (2016) Field calibration of blowfly-derived DNA against traditional methods for assessing mammal diversity in tropical forests. Genome 59:1008–1022
3. Schnell IB, Sollmann R, Calvignac-Spencer S, Siddall ME, Douglas WY, Wilting A, Gilbert MTP (2015) iDNA from terrestrial haematophagous leeches as a wildlife surveying and monitoring tool--prospects, pitfalls and avenues to be developed. Front Zool 12:24
4. Schnell IB, Thomsen PF, Wilkinson N, Rasmussen M, Jensen LRD, Willerslev E, Bertelsen MF, Gilbert MTP (2012) Screening mammal biodiversity using DNA from leeches. Curr Biol 22:R262–3
5. Liedigk R, Kolleck J, Böker KO, et al (2015) Mitogenomic phylogeny of the common long-tailed macaque (Macaca fascicularis fascicularis). BMC Genomics 16:222
6. Patel RP, Förster DW, Kitchener AC, et al (2016) Two species of Southeast Asian cats in the genus Catopuma with diverging histories: an island endemic forest specialist and a widespread habitat generalist. Open Science 3:160350
7. Roos C, Zinner D, Kubatko LS, et al (2011) Nuclear versus mitochondrial DNA: evidence for hybridization in colobine monkeys. BMC Evol Biol 11:77
8. Liedigk R, Roos C, Brameier M, Zinner D (2014) Mitogenomics of the Old World monkey tribe Papionini. BMC Evol Biol 14:176
9. Fortes GG, Paijmans JLA (2015) Analysis of Whole Mitogenomes from Ancient Samples. Methods Mol Biol 1347:179–195
10. Lindgreen S (2012) AdapterRemoval: easy cleaning of next-generation sequencing reads. BMC Res Notes 5:337
11. Hahn C, Bachmann L, Chevreux B (2013) Reconstructing mitochondrial genomes directly from genomic next-generation sequencing reads—a baiting and iterative mapping approach. Nucleic Acids Res.
12. Schubert M, Ermini L, Der Sarkissian C, et al (2014) Characterization of ancient and modern genomes by SNP detection and phylogenomic and metagenomic analysis using PALEOMIX. Nat

Protoc 9:1056–1082

13. Li H, Durbin R (2009) Fast and accurate short read alignment with Burrows–Wheeler transform. Bioinformatics 25:1754–1760
14. McKenna A, Hanna M, Banks E, et al (2010) The Genome Analysis Toolkit: a MapReduce framework for analyzing next-generation DNA sequencing data. Genome Res 20:1297–1303
15. Xie Y, Wu G, Tang J, et al (2014) SOAPdenovo-Trans: de novo transcriptome assembly with short RNA-Seq reads. Bioinformatics 30:1660–1666
16. Lowe TM, Eddy SR (1997) tRNAscan-SE: a program for improved detection of transfer RNA genes in genomic sequence. Nucleic Acids Res 25:955–964
17. Camacho C, Coulouris G, Avagyan V, Ma N, Papadopoulos J, Bealer K, Madden TL (2009) BLAST+: architecture and applications. BMC Bioinformatics 10:421
18. Martin M (2011) Cutadapt removes adapter sequences from high-throughput sequencing reads. EMBnet.journal 17:10–12
19. Gouy M, Guindon S, Gascuel O (2010) SeaView version 4: A multiplatform graphical user interface for sequence alignment and phylogenetic tree building. Mol Biol Evol 27:221–224
20. Wyman SK, Jansen RK, Boore JL (2004) Automatic annotation of organellar genomes with DOGMA. Bioinformatics 20:3252–3255
21. Milne I, Stephen G, Bayer M, Cock PJA, Pritchard L, Cardle L, Shaw PD, Marshall D (2012) Using Tablet for visual exploration of second-generation sequencing data. Brief Bioinform bbs012
22. Katoh K, Standley DM (2013) MAFFT multiple sequence alignment software version 7: improvements in performance and usability. Mol Biol Evol 30:772–780
23. Stamatakis A (2014) RAxML version 8: a tool for phylogenetic analysis and post-analysis of large phylogenies. Bioinformatics 30:1312–1313

Tables and Figures

1. Table 1: List of mitogenomes assembled in this work that supplement pre-existing mitogenome references already available in GenBank
2. Table 2: List of mitogenomes assembled in this work that have no previous complete mitogenome reference available in GenBank
3. Figure 1: Geographical distribution of mitogenomes assembled in this work (60 mitogenomes with known locality)
4. Figure 2: Phylogenetic tree of mitogenomes assembled in this work

Additional files

1. Additional file 1: DNA extraction of historical samples, library construction and primer information
2. Additional file 2: Sample information sheet of mitogenomes assembled in this work
3. Additional file 3: Alignment of mitogenomes assembled in this work
4. Additional file 4: Newick file for phylogenetic tree

Table 1- List of mitogenomes assembled in this work that supplement pre-existing mitogenome references already available

Click here to download Table 1 Table 1.xlsx

Table 1: List of mitogenomes assembled in this work that supplement pre-existing mitogenome references already available in GenBank

No	GenBank ID	Common name	Genus	Species	Assembly Size	Locality	Source	Sample date of collection	Data by
1	KY117537	Hog deer	*Axis*	*porcinus*	16402	Copenhagen Zoo	ZM, KU	21/8/1912	FMS/FP
3	KX265095	Bay Cat	*Catopuma*	*badia*	16960	Sabah, Malaysia	National Museum Scotland	20/04/2000	PRP
5	KY117545	Sumatran Rhino	*Dicerorhinus*	*sumatrensis*	16466	Sumatra, Indonesia	Naturalis, Leiden, The Netherlands	1880	RM
6	KY117546	Least pygmy squirrel	*Exilisciurus*	*exilis*	16637	Indonesia	ROM	16/06/1993	FMS/FP
7	KY117548	Hose's mongoose	*Herpestes*	*javanicus*	16340	Java, Indonesia	ZM, KU	12/3/1947	FMS/FP
8	KY117550	Three-striped ground Squirrel	*Lariscus*	*indsignis*	16399	Maybe Malaysia	ZM, KU	unknown	FMS/FP
9	KY117592	Black Crested Macaque	*Macaca*	*nigra*	16558	captive	Gettorf Zoo, Germany	18/07/2000	CR
10	KY117593	Northern Pig-tailed Macaque	*Macaca*	*leonina*	16554	captive	Ludwig-Maximilans-University Munich, Germany	6/3/1995	CR
11	KY117594	Southern pig-tailed macaque	*Macaca*	*nemestrina*	16531	Peninsular Malaysia	National Museum Scotland, Edinburgh, UK	unknown	CR
12	KT288227	Marbled Cat	*Pardofelis*	*marmorata*	17218	Sumatra, Indonesia	National Archaeological Museum of the Netherlands, Leiden	30/08/1930	PRP
13	KY117602	Sumatra Surili	*Presbytis*	*melalophos*	16558	captive	Howletts Wild Animal Park, UK	23/7/1999	CR
14	KR135743	Flat-headed Cat	*Prionailurus*	*planiceps*	17704	Sabah, Malaysia	Sabah wildlife department	25/04/2000	PRP
15	KY117580	Malayan Field Rat	*Rattus*	*tiomanicus*	16415	SPF Bidor, Perak, Malaysia	FRIM	12/2/2011	FMS/FP
16	KY117579	Malayan Field Rat	*Rattus*	*tiomanicus*	16312	Indonesia	ROM	01/06/1993	FMS/FP
17	KY117581	Malayan Field Rat	*Rattus*	*tiomanicus*	16305	Hutan Simpan Chikus, Tapah Perak, Malaysia	FRIM	13/1/2011	FMS/FP
18	KY117574	Javan Rhino	*Rhinoceros*	*sondaicus*	16417	Java, Indonesia	Copenhagen Natural History Museum	unknown	RM
20	KY117575	Javan Rusa	*Rusa*	*timorensis*	16437	Toeloeng Agoeng, West Java, Indonesia	Naturalis, Leiden, The Netherlands	unknown	RM

21	KY117576	Indian Sambar Deer	*Rusa*	*unicolor dejeani*	16437	Mentawai, Indonesia	Naturalis, Leiden, The Netherlands	unknown	RM
22	KY117599	Western Purple-faced Langur	*Semnopithecu s*	*vetulus*	16545	captive	Belfast Zoo, UK	9/11/1998	CR
23	KY117589	Malayan Tapir	*Tapirus*	*indicus*	16794	captive	Copenhagen Zoo	11/1/2015	FMS/FP
24	KY117598	Silvered Langur	*Trachypithecu s*	*cristatus*	16551	North Sumatra, Indonesia	Bavarian State Collection Munich, Germany	1911	CR

ZM, KU: Zoological Museum, University of Copenhagen; ROM: Royal Ontario Museum; FRIM: Forest Research Institute, Malaysia

Table 2- List of mitogenomes assembled in this work that have no previous complete mitogenome reference available in GenBank

Click here to download Table 2 Table 2.xlsx

Table 2: List of mitogenomes assembled in this work that have no previous complete mitogenome reference available in GenBank

No	GenBank ID	Common name	Genus	Species	Assembly Size	Locality	Source	Sample date of collection	Data by
1	KY117536	Asian Small-clawed Otter	*Aonyx*	*cinereus*	16153	captive	Copenhagen Zoo	08/08/11	FMS/FP
2	KY117535	Asian Small-clawed Otter	*Aonyx*	*cinereus*	16153	Sarawak, Malaysia	British Museum of Natural History, London	25/8/2010	FMS/FP
3	KY117560	Binturong	*Arctictis*	*binturong*	17067	unknown	Tierpark, Berlin	29/11/2010	PRP
4	KY117541	Plantain Squirrel	*Callosciurus*	*notatus*	16582	Hutan Bidor, Perak, Malaysia	FRIM	11/2/2011	FMS/FP
5	KY117542	Plantain squirrel	*Callosciurus*	*notatus*	16599	East Kalimantan, Indonesia	ROM	03/06/1993	FMS/FP
6	KY117543	Prevost's squirrel	*Callosciurus*	*prevostii*	16674	East Kalimantan, Indonesia	ROM	15/06/1993	FMS/FP
7	KY117540	Variable squirrel	*Callosciurus*	*finlaysonii frandseni*	15747	Koh Chang, Thailand	ZM, KU	14/1/1900	FMS/FP
8	KY117539	Variable squirrel	*Callosciurus*	*finlaysonii*	16489	Central Thailand	ZM, KU	2/2/1928	FMS/FP
9	KY117544	Sunda Otter Civet	*Cynogale*	*bennetti*	15784	Borneo	British Museum of Natural History, London	25/8/2010	FMS/FP
10	KY117549	Greater Mouse deer	*Tragulus*	*napu*	15778	Bang Nara, Thailand	ZM, KU	10.11.1931	FMS/FP
11	KY117552	Long-tailed Giant Rat	*Leopaldamys*	*sabanus*	15973	G. Telapak Buruk, Negeri Sembilan, Malaysia	FRIM	24/2/2010	FMS/FP
12	KY117553	Long-tailed Giant Rat	*Leopaldamys*	*sabanus*	15972	Teluk Segadas, P. Pangkor, Perak, Malaysia	FRIM	19/3/2010	FMS/FP
13	KY117554	Long-tailed Giant Rat	*Leopaldamys*	*sabanus*	15974	Hutan Simpan Temengor,Gerik Perak, Malaysia	FRIM	23/1/2014	FMS/FP
14	KY117555	Long-tailed Giant Rat	*Leopaldamys*	*sabanus*	15972	Hutan Simpan Lenggor, Kluang, Johor, Malaysia	FRIM	19/2/2014	FMS/FP
15	KY117551	Long-tailed giant rat	*Leopaldamys*	*sabanus*	15974	Malaysia	ROM	28/05/1993	FMS/FP
16	KY117556	Hairy-nosed otter	*Lutra*	*sumatrana*	16580	Bang Nara, Thailand	ZM, KU	1/4/1939	FMS/FP
17	KY117557	Smooth-coated otter	*Lutrogale*	*perspicillata*	16042	Melaka, Malaysia	British Museum of Natural History, London	25/8/2010	FMS/FP

18	KY117558	Smooth-coated otter	*Lutrogale*	*perspicillata*	16041	Bang Nara, Thailand	ZM, KU	24/1/1933	FMS/FP
19	KY117591	Moor macaque	*Macaca*	*maura*	16563	captive	Hannover Zoo, Germany	20/8/1998	CR
20	KY117564	Rajah/Brown Spiny Rat	*Maxomys*	*rajah*	16200	Indonesia	ROM	06/06/1993	FMS/FP
21	KY117562	Rajah/Brown Spiny Rat	*Maxomys*	*rajah*	16296	Teluk Segadas, P. Pangkor, Perak, Malaysia	FRIM	19/3/2010	FMS/FP
22	KY117563	Rajah/Brown Spiny Rat	*Maxomys*	*rajah*	16296	Pasir Bogak, P.Pangkor, Perak, Malaysia	FRIM	18/3/2010	FMS/FP
23	KY117567	Red Spiny Rat	*Maxomys*	*surifer*	16286	50 ha, Pasoh, Negeri Sembilan, Malaysia	FRIM	12/6/2008	FMS/FP
24	KY117566	Red Spiny Rat	*Maxomys*	*surifer*	16290	Indonesia	ROM	21/05/1993	FMS/FP
25	KY117565	Red Spiny Rat	*Maxomys*	*surifer*	16286	Malaysia	ROM	17/05/2013	FMS/FP
26	KY117570	Whitehead's Spiny Rat	*Maxomys*	*whiteheadi*	16316	Hutan Simpan Bikam, Perak, Malaysia	FRIM	12/2/2011	FMS/FP
27	KY117571	Whitehead's Spiny Rat	*Maxomys*	*whiteheadi*	16316	Keruing Trail, FRIM, Kepong, Selangor, Malaysia	FRIM	13/3/2013	FMS/FP
28	KY117568	Whitehead's Spiny Rat	*Maxomys*	*whiteheadi*	16287	Hutan Simpan Bikam, Perak, Malaysia	FRIM	12/2/2011	FMS/FP
29	KY117569	Whitehead's Spiny Rat	*Maxomys*	*whiteheadi*	16429	Bukit Tapah, Perak, Malaysia	FRIM	23/3/2011	FMS/FP
30	KY052142	Indian muntjac	*Muntiacus*	*muntjak*	16354	West Java, Indonesia	Vienna NHM	1858	RM
31	KY117559	Bornean yellow muntjac	*Muntiacus*	*atherodes*	16354	Koemai, West Borneo	Bonn NHM	1938	RM
32	KY117573	Dark-tailed Tree Rat	*Niviventer*	*cremoriventer*	16322	Track 5 (G.Inas), Kedah, Malaysia	FRIM	5/11/2009	FMS/FP
33	KY117572	Dark-tailed tree rat	*Niviventer*	*cremoriventer*	16234	Malaysia	ROM	17/05/2013	FMS/FP
34	KY117600	Grizzled Leaf Monkey	*Presbytis*	*comata comata*	16551	captive	Howletts Wild Animal Park, UK	23/12/1999	CR
35	KY117601	Mitred Leaf Monkey	*Presbytis*	*mitrata*	16555	captive	Howletts Wild Animal Park, UK	12/11/1998	CR
36	KX857784	Leopard cat	*Prionailurus*	*bengalensis*	16989	Thailand	American Museum of National History, New York.	25/02/1924	PRP

37	KY117578	Annandale’s Sundaic Rat	*Rattus*	*annandalei*	16297	Hutan Simpan Bikam, Perak, Malaysia	FRIM	12/2/2011	FMS/FP
38	KY117577	Annandale’s Sundaic Rat	*Rattus*	*annandalei*	16301	Hutan Simpan Bikam, Perak, Malaysia	FRIM	11/2/2011	FMS/FP
39	KY117583	Mountain giant Sunda rat	*Sundamys*	*infraluteus*	16297	Malaysia	ROM	18/05/2013	FMS/FP
40	KY117585	Müller's Giant Sunda Rat	*Sundamys*	*meulleri*	16326	Track 1 (G.Inas), Kedah, Malaysia	FRIM	5/11/2009	FMS/FP
41	KY117584	Müller's giant Sunda rat	*Sundamys*	*meulleri*	16304	Malaysia	ROM	01/06/2013	FMS/FP
42	KY117586	Brooke's squirrel	*Sundasciurus*	*brookei*	16417	East Kalimantan, Indonesia	ROM	13/06/1993	FMS/FP
43	KY117587	Low's squirrel	*Sundasciurus*	*lowii*	16307	East Kalimantan, Indonesia	ROM	06/06/1993	FMS/FP
44	KY117588	name pending	*Sundasciurus*	*sp*	16458	East Kalimantan, Indonesia	ROM	21/06/1993	FMS/FP
45	KY117595	Phayre's Langur	*Trachypithecus*	*phayrei phayrei*	16548	South West Myanmar	Natural History Museum Berlin, Germany	unknown	CR
46	KY117596	East Javan Ebony Langur	*Trachypithecus*	*auratus*	16552	captive	Bristol Zoo, UK	26/10/2010	CR
47	KY117597	West Javan Ebony Langur	*Trachypithecus*	*mauritius*	16554	West Java , Indonesia	Naturalis Leiden; Netherlands	unknown	CR
48	KY117590	Long-tailed porcupine	*Trichys*	*fasciculata*	16328	Borneo	ZM, KU	5/10/1894	FMS/FP

ZM, KU: Zoological Museum, University of Copenhagen; ROM: Royal Ontario Museum; FRIM: Forest Research Institute, Malaysia; NHM: Natural History Museum

Figure 1 Geographical distribution of mitogenomes assembled in this work (60 mitogenomes with known locality)

Click here to download Figure 4 Figure 1.pdf

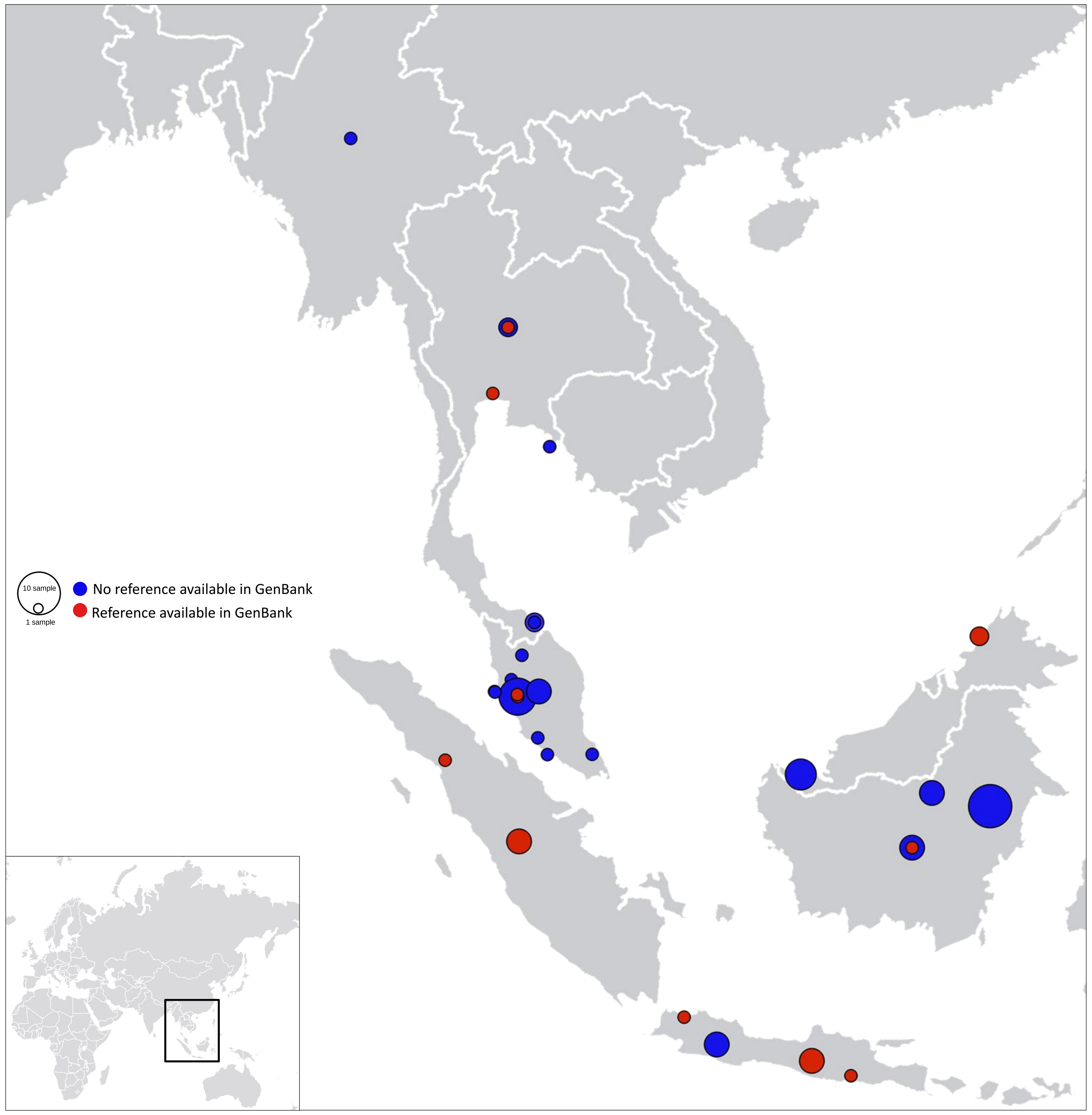

Figure 1 Geographical distribution of mitogenomes assembled in this work (60 mitogenomes with known locality)

Click here to download Figure 4 Figure 1.png

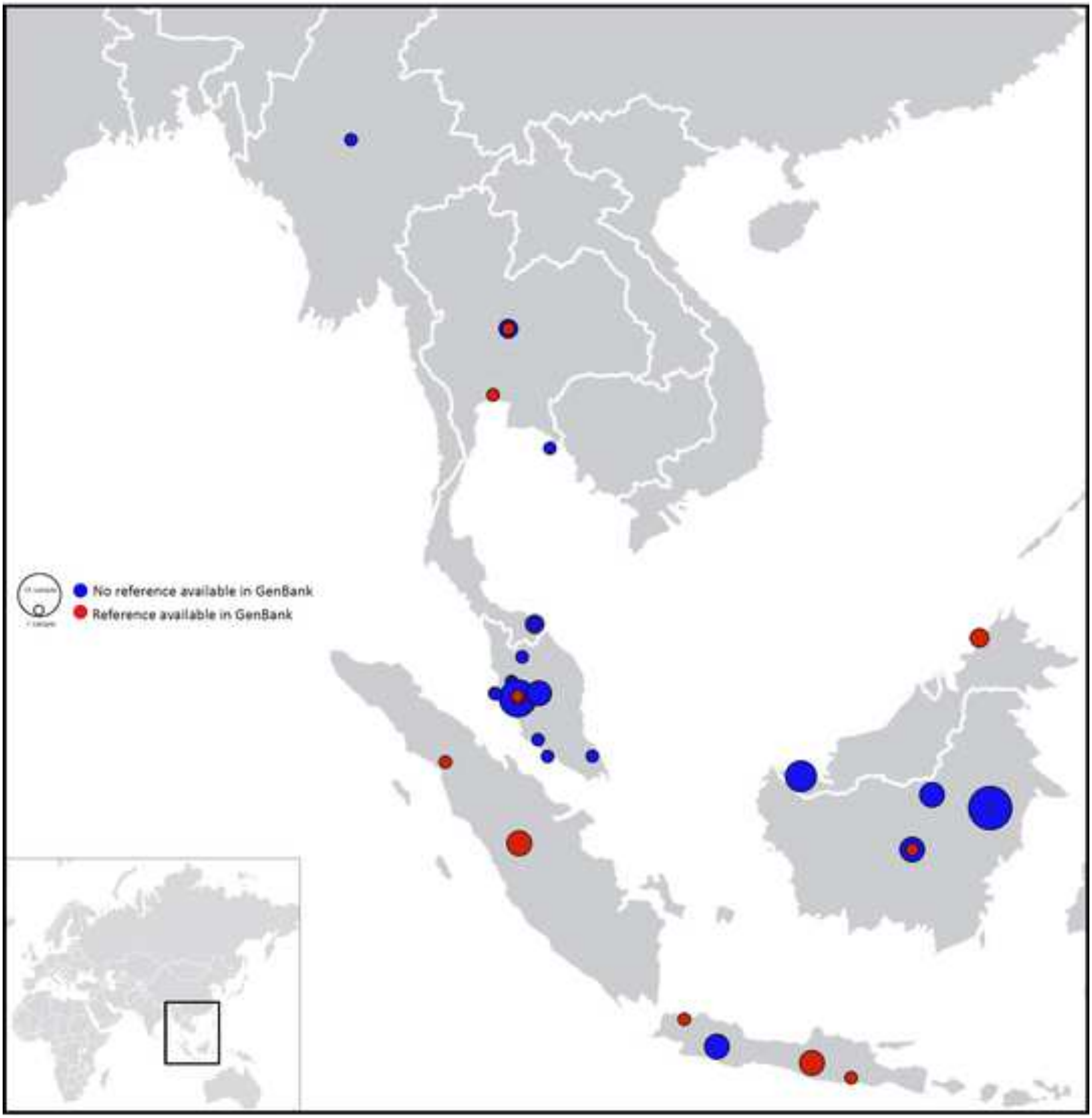

Figure 2 Phylomitogenetic tree of mitogenomes assembled in this work

Click here to download Figure 4 Figure 2.pdf

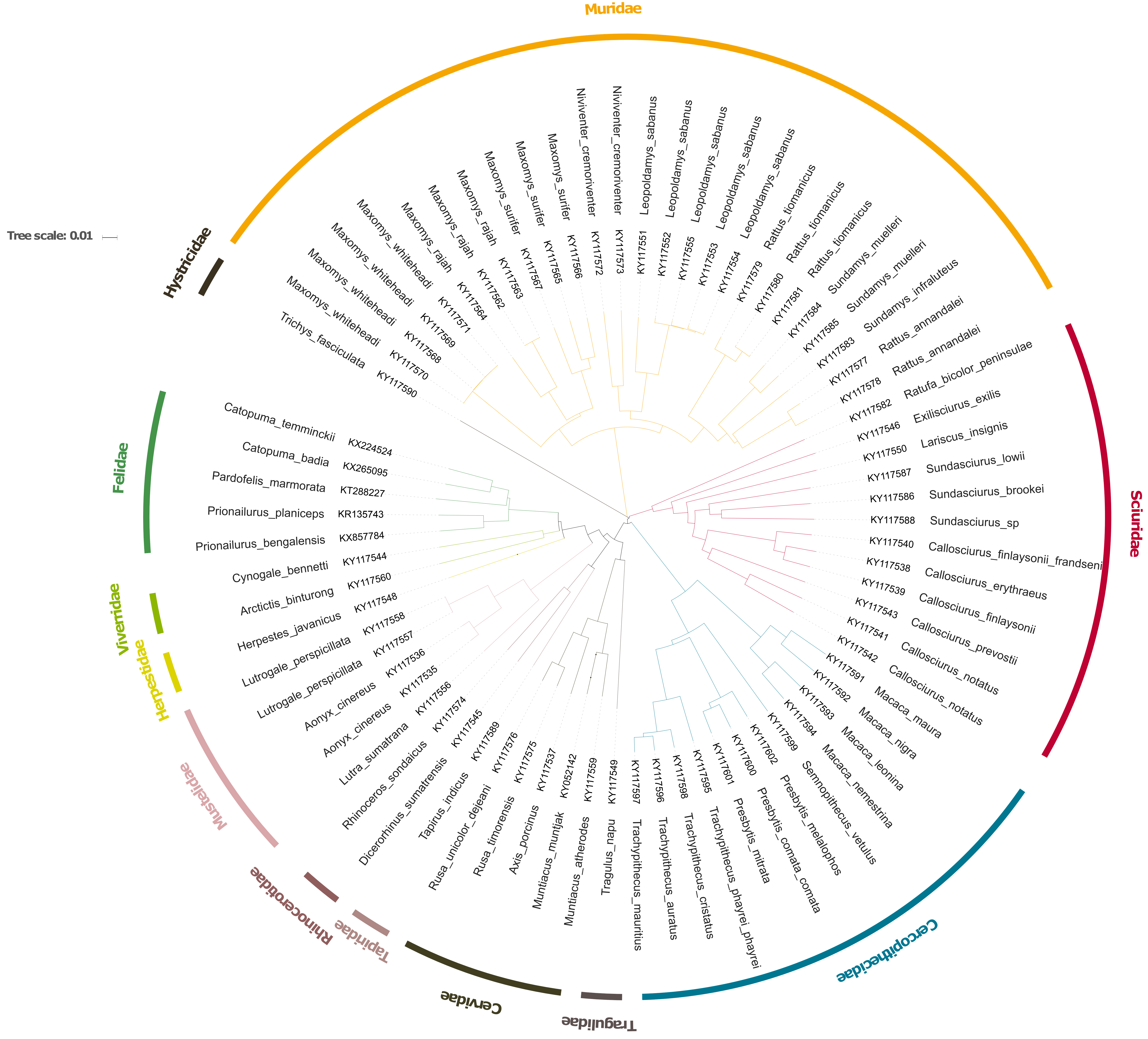

README file salleh et al

Click here to access/download
Supplementary Material
READMEsallehetal.txt

Data Information salleh et al

Click here to access/download
Supplementary Material
1DatainformationSallehetal.docx

Additional file 2 Sample information sheet of 72 SE asian small mammal mitogenomes

Click here to access/download
Supplementary Material
5 Additional file 2.xlsx

Additional file 3 Alignment of mitogenomes assembled in this work

Click here to access/download
Supplementary Material
5 Additional file 3.fa

Additional file 4 newick

Click here to access/download
Supplementary Material
5 Additional file 4 newick.txt

Additional file 1 DNA extraction for historical samples and primer information

Click here to access/download
Supplementary Material
5Additional file 1.docx

Click here to access/download
Supplementary Material
Correctionsforsubmissions.docx
