# Supplementary material for: An expanded mammal mitogenome dataset from Southeast Asia
Source: Gigascience. 2017 Jul 13;6(8):1–8. doi: 10.1093/gigascience/gix053 (PMC5737531; doi:10.1093/gigascience/gix053)
Supplement: Additional-files [file gix053_supp.zip › 5Additional file 1_CR 150617.docx]

**Additional file 1a:** DNA extraction for historical samples obtained from the Zoological Museum, Natural History Museum of Denmark, University of Copenhagen, Denmark.

The historical samples were processed in an ancient DNA (aDNA) laboratory at the University of Copenhagen, Denmark following standard practices in aDNA research to minimize contamination. This includes the usage of full body suits, nightly UV irradiation of the laboratory, frequent bleaching of surfaces, and physical separation between pre- and post-PCR areas.

Prior to extraction, bone samples were pulverized with a Braun Mikro Dismembrator S ball mill (B. Braun Biotech, Melsungen, Germany) using a stainless steel flask and grinding ball. Prior to grinding both the flask and grinding ball were sterilized with a 5% solution of commercial sodium hypochlorite solution (bleach), followed by rinsing with DNA AWAY (Molecular BioProducts, San Diego, CA), and finally UV-irradiated for 5 minutes. The pulverization was done at 2000 RPM for 20 seconds. Skin samples were cut into small (ca 5 mm^2^) pieces and were individually vortexed in a 10% commercial sodium hypochlorite solution (bleach) solution to decontaminate surface. In order to remove the bleach, the samples were subsequently vortexed in 70% ethanol and finally vortexed in sterilized water.

The crushed bone powder and treated skin samples were then added to 1 mL digestion buffer consisting of 10 mM tris(hydroxymethyl)aminomethane hydrochloride (Tris-HCl) (pH 8.0), 10 mM NaCl, 2% w/v sodium dodecyl sulfate (SDS), 5 mM CaCl2, 2.5 mM ethylenediaminetetraacetic acid (EDTA) (pH 8.0), 40 mM dithiothreitol (DTT), and 10% Proteinase K [[1]](https://paperpile.com/c/2SRZo6/5Axf). The samples were incubated for 12 hours at 56°C. In order to purify the DNA from contaminants, 1mL supernatant was mixed with 1mL phenol, vortexed for 20 sec, gently rotated for 5 min and centrifuged at 3000 g for 3 min. Approximately 1mL aqueous liquid was removed and mixed with 1mL chloroform by vortexing for 30 sec. The mixture was following rotated for 5 min and centrifuged at 3000 g for 3 min. Approximately 1mL aqueous liquid was removed and purified using the MinElute PCR Purification kit (Qiagen, Valencia, CA) according to manufacturer’s instruction with slight modification: firstly, the PB buffer was modified according to [[2]](https://paperpile.com/c/2SRZo6/kGRd). Secondly, the volume of PB binding buffer was increased to 10x. The buffer was applied to the spin columns following the method developed by [[3]](https://paperpile.com/c/2SRZo6/P6zo), a Zymo-Spin V reservoir (Zymo Research, Irvine, CA) was used to pass the large buffer volume through the MinElute column. Prior to the final centrifugation, a repeated step of adding 15 μL of EB buffer to the column and incubated for 15 minutes at 37°C, centrifugation at 6000 g for 1 min. The extracted DNA was quantified using a Qubit fluorometer with a dsDNA high sensitivity (HS) assay (Life Technologies, Carlsbad, CA).

**Additional file 1b:** Library construction details for FMS mitogenomes

FMS mitogenomes were generated through mining of mitochondrial DNA reads generated using Illumina HiSeq shotgun sequence data, following initial conversion of the DNA extract into an Illumina-compatible sequencing library using Meyer and Kirscher’s blunt end protocol [[4]](https://paperpile.com/c/2SRZo6/jRxn), although modified to be compatible with the commercial NEBNext E6070 kit (New England Biolabs, Ipswich, MA). The library was PCR amplified and indexed in 100 μL reactions, with each reaction containing 20 μL of template DNA, 10 U AmpliTaq Gold polymerase (Applied Biosystems, Foster City, CA), 1×AmpliTaq Gold buffer, 2.5 mM MgCl2, 0.2 mM of each dNTP, 0.2 uM IS4 forward primer, 0.2 uM reverse primer with 6 bp index, and 0.4 mg/mL bovine serum albumin. The PCR conditions were 12 minutes at 95°C to denature DNA and activate the polymerase, 12 cycles of 95°C for 20 seconds, 60°C annealing for 30 seconds, and 72°C extension for 40 seconds, and a final extension of 72°C extension for 5 minutes. Following amplification, the library was purified using a QIAquick PCR Purification kit (Qiagen), according to manufacturer’s directions, and DNA was eluted in 30 μL. Quantification and size estimation of the library was performed on a Bioanalyzer 2100 High Sensitivity DNA chip (Agilent Technologies, Santa Clara, USA). Multiple libraries were combined together into three pools, normalized to 10 nM and sequenced on three lanes of Illumina HiSeq 2500 using SR100bp chemistry.

**Additional file 1c:** Primer details for PRP mitogenomes

For sample 3 (*Arctictis binturong),* PRP used primer combination of three overlapping DNA fragments which covered the *A. binturong* mitochondrial genome (designed with Geneious 6.1 (Geneious® 6.1.7, 2013) using the following genes: NAD2, NAD4 and control region (D-loop) of *A. binturong* available in Genbank. Primer details are as as below. Long range PCR was followed using MyFiTM Mix Polymerase (Bioline GmbH, Germany), and an expanded elongation time (8min) ensured the amplification of at least 8000 base pairs. For sample 11, 12, 48, 52 and 53, primers for three large (~6kb) overlapping regions of the leopard cat (*Prionailurus bengalensis*) mitogenome were designed using Genbank reference mitogenome NC_016189 in Geneious 6.1 (Geneious® 6.1.7,2013). Primer details and long-range PCR protocol are described in [[5]](https://paperpile.com/c/2SRZo6/PFt2) and (Patel et. al. in review), respectively. These long range PCR products were subsequently sheared to ~250bp using a Covaris M220 (Covaris Inc., USA), purified using the QiaQuick kit (Qiagen), and pooled equimolarly. Pooled PCR products were then used as template to prepare Illumina library.

Primers for generating mitogenomes sample 3: *A. binturong.* A combination of six primers (designed with Geneious 6.1 (Geneious® 6.1.7, 2013) using the following genes: NAD2, NAD4 and control region (D-loop) of *A. binturong* available in Genbank) were used to generate three overlapping DNA fragments which covered the *A. binturong* mitochondrial genome.

| **Primer ID** | **gene** | **Sequence** |
| --- | --- | --- |
| ABI F1_F | Control region | CAAGGAAGAAGCAACAGCCC |
| ABI F1_R | Control region | TGGGTTCAGTCCTCCCTCAT |
| ABI F2_F | NAD2 | CCCACGAGCCATAGAAGCTT |
| ABI F2_R | NAD2 | CGTGGGCGATTATTAGGGCT |
| ABI F3_F | NAD4 | TGACTACCCAAAGCGCATGT |
| ABI F3_R | NAD4 | GCTAACGGTTCATGTACGGTG |

**Additional File 1d:** Sanger sequencing details for CR mitogenomes

Complete mitogenomes from 12 primates were amplified from DNA extracted from blood, fresh tissue or museum samples either via 2 long range PCR with product sizes of 8-10 kb and an overlap of 500-800 bp followed by nested PCR or as in the case of museum material directly via 21 PCRs with product sizes of 1.0-1.2 kb and an overlap of 100-300 bp. All working steps (DNA extraction, PCR setup, gel electrophoresis, PCR product purification, sequencing) were conducted in separate laboratories and under Captair Bio PCR cabinets to prevent cross-sample contamination. Museum samples were handled in the in-house ancient DNA laboratory. Further, negative PCR controls (without template DNA) were routinely conducted. Long range PCRs were performed with the Expand Long Range dNTPack (Roche) following the supplier’s recommendations for PCR set-up and cycling conditions. Only annealing temperatures were adjusted. Primer sequences are available upon request. PCR products were run on 1% agarose gels and excised from the gel. After purification with the Qiagen Gel Extraction Kit, long range PCR products were subjected to nested PCR. The 21 nested PCRs or the direct amplification of mitogenomes via 21 PCRs from museum material was carried out in a total volume of 30µl containing 1 U BiothermTaq 5000 (Genecraft), 1X reaction buffer, 0.16 mM of each dNTP, 0.33 μM of each primer, 0.6 mg/ml BSA and 10-50 ng template. Thermo cycler conditions comprised of 94°C for 2 min, followed by 40-50 cycles of 94°C for 1 min, 50-60°C for 1 min and 72°C for 1.5 min, followed by 72°C for 5 min. PCR performance and product sizes were again checked on 1% agarose gels, and after purification with the Qiagen PCR Purification Kit, PCR products were sequenced on an ABI 3130xL sequencer using the BigDye Terminator 3.1 Cycle Sequencing kit (Applied Biosystems) and both amplification primers.

1. [Gilbert MTP, Tomsho LP, Rendulic S, et al (2007) Whole-genome shotgun sequencing of mitochondria from ancient hair shafts. Science 317:1927–1930](http://paperpile.com/b/2SRZo6/5Axf)

2. [Allentoft ME, Sikora M, Sjögren K-G, et al (2015) Population genomics of Bronze Age Eurasia. Nature 522:167–172](http://paperpile.com/b/2SRZo6/kGRd)

3. [Dabney J, Knapp M, Glocke I, et al (2013) Complete mitochondrial genome sequence of a Middle Pleistocene cave bear reconstructed from ultrashort DNA fragments. Proc Natl Acad Sci U S A 110:15758–15763](http://paperpile.com/b/2SRZo6/P6zo)

4. [Meyer M, Kircher M (2010) Illumina sequencing library preparation for highly multiplexed target capture and sequencing. Cold Spring Harb Protoc 2010:db.prot5448](http://paperpile.com/b/2SRZo6/jRxn)

5. [Patel RP, Förster DW, Kitchener AC, et al (2016) Two species of Southeast Asian cats in the genus Catopuma with diverging histories: an island endemic forest specialist and a widespread habitat generalist. Open Science 3:160350](http://paperpile.com/b/2SRZo6/PFt2)
