# Supplementary material for: An expanded mammal mitogenome dataset from Southeast Asia
Source: Gigascience. 2017 Jul 13;6(8):1–8. doi: 10.1093/gigascience/gix053 (PMC5737531; doi:10.1093/gigascience/gix053)
Supplement: Response-to-Reviewer-Comments_Original-Submission.pdf [file gix053_response-to-reviewer-comments_original-submission.pdf]

Author's Response To Reviewer Comments

Reviewer reports:
Reviewer #2: Douglas Yu
This paper should be published, and the mitogenomes will come in handy. I did, however, check genbank for the assemblies, but they are mostly not available (the two Catopuma assemblies are available but under a separate paper). are the assemblies under publication embargo? I have no other comments.
Yes, the data is currently still under publication embargo.

Reviewer #1: John-James Wilson
Thank you for this opportunity to review a GigaScience Data Note.
This is an important dataset (as the authors nicely explain) with a vast range of potential usages. In particular, this dataset could be an invaluable resource to the application of mammal monitoring via eDNA and iDNA approaches in Southeast Asia, which is an applied research area in which my lab has done some preliminary work [Genome, 2016, 59(11): 1008-1022, 10.1139/gen-2015-0193].
The data all appears to be readily available and has been generated following standard protocols from a variety of collections and labs from around the world. As such, this Data Note represents an impressive collaboration. Given that this is largely novel data, any discrepancies in terms of species identifications etc., will only become apparent as more similar data accumulates, and through incorporation of this data into future analyses. Therefore the release of this data is commendable.

Consequently, my comments are restricted to minor issues regarding the context of the data and the presentation of the Data Note.

Abstract
- The title and abstract highlight Southeast Asia, but samples were obtained from localities as geographically widespread as Sri Lanka (South Asia) and Taiwan (East Asia). While these samples may represent species also found in Southeast Asia (?), it is possible they could be quite divergent (in their mitochondrial sequences) from conspecifics from Southeast Asia proper. In any case, it would be good to justify how the selection of samples obtained for mitogenome sequencing was done, if the authors want to retain the emphasis on this geographic region.

We have removed the three samples below, now making the total mitogenomes 72.

Genebank ID KY117561 Muntiacus reevesi Shin-Yi, Taiwan
Genebank ID KY117547 Herpestes hosei Ceylon
Genebank ID KY052092 Muntiacus vaginalis Gangtok, South Sikkim, India

- Mitochondrion barcodes or mitochondrial barcodes?
Corrected to mitochondrial barcodes

- The phase "we have contributed to this need" is a bit confusing. A better phrase may be "we have partially addressed this need".
corrected

- It may be clearer to say the 32 species had no previous mitogenome available rather than have no current mitogenome available.
corrected

- It would be good to say how mammal species from SEA now have mitogenomes available. What percentage do the 32 novel mitogenomes from this study contribute?
Currently there are 922 mammalian mitogenomes available in Genbank. Unfortunately most are not tagged by location/origin. Data mining through manual screening of each mitogenomes resulted in 174 terrestrial mammal species which are typical to SE Asia. In this work, 30 novel species are added, contributing to ~17% expansion of the current SE Asia mammal mitogenome database.
You can view the list in the link below:
https://docs.google.com/spreadsheets/d/1t3pkxCMKbogQjX5YfLGW1waDaD9NL1_eMYzI5CdFoJo/edit#gid=611706729

- I'm not sure why Malaysia is a keyword given the broad range of countries from which samples were obtained?
removed

Data description
-The context is a bit brief, but may be adequate for a Data Note?

-Could GPS coordinates be given in table 1 and 2? I would find this more useful than the map.
The GPS locations are given in Additional file 2. We decided not to add them in Tables 1 and 2 because most samples, especially museum specimens don't have their exact location documented (e.g Sumatra). The GPS presents a rough location with accuracy around 100 km. Thus, we thought the map would be better to show the mitogenome distribution in this work.

-In the tables I suggest not to italicise the vernacular English name, to avoid confusion with scientific names.
corrected

-In the tables, in the assembly size column, sometimes a comma is used and other times not.
corrected

-Museum is miss-spelled in the table footers and in other places in the tables.
corrected

-In my experience West Malaysia is most commonly referred to as Peninsular Malaysia. It would be good to include states and provinces in the localities if known. Also, it would be good to be

consistent and always put the country name i.e. Sumatra, Indonesia. Besides, a strange spelling is used for Malacca/Melaka.
corrected

-Central Siam is present-day Thailand? Ceylon is Sri Lanka? In general it would be good to convert all country/state/city names to their currently used forms. The specimen/museum label form of the locality could be given in another column in the table or in parentheses if necessary.
corrected

-For the DNA extraction protocols (which are given very briefly) it would be good to state that these are standard protocols- that they were performed following the manufacturer's guidelines or following given references etc. I know reference are given in a few cases, but not all.
corrected

-I'm not sure what the phylogenetic analysis contributes to this Data Note.
It shows a clearer taxonomical order distribution of mitogenomes presented in this work.
