# Supplementary material for: An expanded mammal mitogenome dataset from Southeast Asia
Source: Gigascience. 2017 Jul 13;6(8):1–8. doi: 10.1093/gigascience/gix053 (PMC5737531; doi:10.1093/gigascience/gix053)
Supplement: Response-to-Reviewer-Comments_Revision-1.pdf [file gix053_response-to-reviewer-comments_revision-1.pdf]

Author's Response To Reviewer Comments

1) Add Re-use potential section at the end of the main text.
-Added.

2) Move all URLs in the main text to the references list (see comments in manuscript attached)
-Done.

3) Please add funding information where indicated.
-Done
