# Supplementary material for: An expanded mammal mitogenome dataset from Southeast Asia
Source: Gigascience. 2017 Jul 13;6(8):1–8. doi: 10.1093/gigascience/gix053 (PMC5737531; doi:10.1093/gigascience/gix053)
Supplement: Reviewer-1-Report-(Original-Submission).pdf [file gix053_reviewer-1-report-(original-submission).pdf]

Reviewer Report

Title: An expanded mammal mitogenome dataset from Southeast Asia

Version: Original Submission **Date:** 2/17/2017

Reviewer name: John-James Wilson

Reviewer Comments to Author:

Peer-Review Report: An expanded mammal mitogenome dataset from Southeast AsiaThank you for this opportunity to review a GigaScience Data Note.This is an important dataset (as the authors nicely explain) with a vast range of potential usages. In particular, this dataset could be an invaluable resource to the application of mammal monitoring via eDNA and iDNA approaches in Southeast Asia, which is an applied research area in which my lab has done some preliminary work [Genome, 2016, 59(11): 1008-1022, 10.1139/gen-2015-0193].The data all appears to be readily available and has been generated following standard protocols from a variety of collections and labs from around the world. As such, this Data Note represents an impressive collaboration. Given that this is largely novel data, any discrepancies in terms of species identifications etc., will only become apparent as more similar data accumulates, and through incorporation of this data into future analyses. Therefore the release of this data is commendable.Consequently, my comments are restricted to minor issues regarding the context of the data and the presentation of the Data Note.Abstract- The title and abstract highlight Southeast Asia, but samples were obtained from localities as geographically widespread as Sri Lanka (South Asia) and Taiwan (East Asia). While these samples may represent species also found in Southeast Asia (?), it is possible they could be quite divergent (in their mitochondrial sequences) from conspecifics from Southeast Asia proper. In any case, it would be good to justify how the selection of samples obtained for mitogenome sequencing was done, if the authors want to retain the emphasis on this geographic region.- Mitochondrion barcodes or mitochondrial barcodes?- The phase "we have contributed to this need" is a bit confusing. A better phrase may be "we have partially addressed this need".- It may be clearer to say the 32 species had no previous mitogenome available rather than have no current mitogenome available.- It would be good to say how mammal species from SEA now have mitogenomes available. What percentage do the 32 novel mitogenomes from this study contribute?- I'm not sure why Malaysia is a keyword given the broad range of countries from which samples were obtained?Data description-The context is a bit brief, but may be adequate for a Data Note?-Could GPS coordinates be given in table 1 and 2? I would find this more useful than the map.-In the tables I suggest not to italicise the vernacular English name, to avoid confusion with scientific names.-In the tables, in the assembly size column, sometimes a comma is used and other times not.-Museum is miss-spelled in the table footers and in other places in the tables.-In my experience West Malaysia is most commonly referred to as Peninsular Malaysia. It would be good to include states and provinces in the localities if known. Also, it would be good to be consistent and always put the country name i.e. Sumatra, Indonesia. Besides, a strange spelling is used for Malacca/Melaka.-Central Siam is present-day Thailand? Ceylon is Sri Lanka? In general it would be good to convert all country/state/city names to their currently used forms. The specimen/museum label form of the locality could be given in another column in the table or in

parentheses if necessary.-For the DNA extraction protocols (which are given very briefly) it would be good to state that these are standard protocols- that they were performed following the manufacturer's guidelines or following given references etc. I know reference are given in a few cases, but not all.-I'm not sure what the phylogenetic analysis contributes to this Data Note.

Level of Interest

Please indicate how interesting you found the manuscript: An article of importance in its field

Quality of Written English

Please indicate the quality of language in the manuscript: Needs some language corrections before being published

Declaration of Competing Interests

Please complete a declaration of competing interests, considering the following questions:

- Have you in the past five years received reimbursements, fees, funding, or salary from an organisation that may in any way gain or lose financially from the publication of this manuscript, either now or in the future?
- Do you hold any stocks or shares in an organisation that may in any way gain or lose financially from the publication of this manuscript, either now or in the future?
- Do you hold or are you currently applying for any patents relating to the content of the manuscript?
- Have you received reimbursements, fees, funding, or salary from an organization that holds or has applied for patents relating to the content of the manuscript?
- Do you have any other financial competing interests?
- Do you have any non-financial competing interests in relation to this paper?

If you can answer no to all of the above, write 'I declare that I have no competing interests' below. If your reply is yes to any, please give details below.

I declare that I have no competing interests

I agree to the open peer review policy of the journal. I understand that my name will be included on my report to the authors and, if the manuscript is accepted for publication, my named report including any attachments I upload will be posted on the website along with the authors' responses. I agree for my report to be made available under an Open Access Creative Commons CC-BY license (http://creativecommons.org/licenses/by/4.0/). I understand that any comments which I do not wish to be included in my named report can be included as confidential comments to the editors, which will not be published.

I agree to the open peer review policy of the journal
